# Preparation of Ru-Based Systems Through Metal Carbonyl Cluster Decomposition for the Base-Free 5-Hydroxymethylfurfural (HMF) Oxidation

**DOI:** 10.3390/molecules30102120

**Published:** 2025-05-10

**Authors:** Francesca Liuzzi, Francesco Di Renzo, Cristiana Cesari, Alice Mammi, Lorenzo Monti, Alessandro Allegri, Stefano Zacchini, Giuseppe Fornasari, Nikolaos Dimitratos, Stefania Albonetti

**Affiliations:** 1 Department of Industrial Chemistry, C3-Centre for Chemical Catalysis, Alma Mater Studiorum—University of Bologna, 40129 Bologna, Italy; francesca.liuzzi7@unibo.it (F.L.); cristiana.cesari2@unibo.it (C.C.); alice.mammi2@studio.unibo.it (A.M.); lorenzo.monti16@studio.unibo.it (L.M.); alessandro.allegri2@unibo.it (A.A.); stefano.zacchini@unibo.it (S.Z.); giuseppe.fornasari@unibo.it (G.F.); nikolaos.dimitratos@unibo.it (N.D.); 2 ICGM, Université de Montpellier-CNRS-ENSCM, 34293 Montpellier, France; francesco.di-renzo@umontpellier.fr

**Keywords:** biomass valorization, metal carbonyl clusters, heterogeneous catalysis, nanoparticles-based catalysts

## Abstract

Metal carbonyl clusters, which can be seen as monodispersed and atomically defined nanoparticles stabilized by CO ligands, were used to prepare Ru-based catalysts with tuned basic properties to conduct the 5-hydroxymethylfurfural (HMF) aerobic oxidation to produce 2,5-furandicarboxylic acid (FDCA) in base-free conditions. The controlled decomposition of the carbonyl cluster [HRu_3_(CO)_11_]^−^, a methodology not yet applied to Ru catalysts for this reaction, on different supports focusing on controlling and tuning the basic properties of support allowed the formation of small Ru nanoparticles with a mean diameter of around 1 nm. The catalytic systems obtained resulted in more activity in the HMF oxidation than those prepared through a more common salt-impregnation technique, and the deposition of Ru nanoparticles on materials with basic functionalities has allowed avoiding the use of basic solutions in the reaction. The characterization by CO_2_-TPD of Mg(Al)O catalysts obtained from decomposition of layered double hydroxide hydrotalcites with different composition and activation has allowed disclosure of an important correlation between the selectivity of FDCA and the fraction of weak basic sites, which is decreased by the calcination treatment at increased temperature.

## 1. Introduction

Metal carbonyl nanoclusters are effective precursors of supported nanoparticles for heterogeneous catalysis [1,2,3,4]. Molecular clusters appear as perfectly monodisperse and atomically defined metal nanoparticles stabilized by surrounding ligands. The chemistry of ligand-protected molecular metal clusters overlaps with that of ultrasmall metal nanoparticles, sharing the same nanometric dimension. The extensive knowledge, experimental techniques, and methods developed for the preparation, purification, and characterization of molecular metal clusters may play a major role in a better understanding of the deposition of ultrasmall metal nanoparticles [5]. It is especially relevant to understand how the dimensionality of metal nanoclusters changes with the interaction with the support and allows tuning the size of custom-tailored metal nanoparticles for heterogeneous catalysis [6,7,8,9,10,11].

Supported gold- or platinum-based catalysts prepared from metal carbonyls have proved to be effective catalysts for several oxidation reactions [12,13], among them the oxidation of 5-hydroxymethylfurfural (HMF) to produce 2,5-furandicarboxylic acid (FDCA) [14]. In the present manuscript, we have focused on different cluster-derived catalysts for the oxidation of HMF to FDCA, a dicarboxylic acid acknowledged as one of the most promising biobased chemicals, as it can replace diacids obtained from fossil sources in the manufacture of polyesters, polyamides, and polyurethanes [15,16].

Ruthenium is receiving a growing interest among the metals for catalysis, as the cheapest and most available of the platinum group metals (PGM) [17]. It is increasingly accompanying or replacing platinum in fuel cell catalysts and electronic components for random access memories [18]. Ruthenium is indeed much more available than other PGMs in the list of critical raw materials (CRM) of the European Union, being currently produced, nearly exclusively in South Africa, at a rate of about 33 t/y [19]. Its two major applications are as electrode coating for water electrolysis and as a classical staple in hydrogenation homogeneous catalysis [20,21,22].

Ruthenium carbonyl complexes also play an important role in oxidation catalysis, especially as homogeneous catalysts of the aerobic oxidation of alcohols to aldehydes and amines to imines [23,24,25]. Ruthenium complexes have catalyzed the oxidation of aldehydes to carboxylic acids by using water as the formal oxidant [26]. Interesting results have been obtained by anaerobic oxidation of HMF to FDCA by alkaline water in the presence of Ru complexes [27]. In heterogeneous catalysis of oxidation reactions, ruthenium is mainly used as RuO_2_ or by impregnation of supports with Ru^3+^ solutions [28,29,30]. Ru(OH)x catalysts for the oxidation of HMF to FDCA were prepared by deposition of RuCl_3_ on several supports [31,32,33]. Even when Ru metal nanoparticles (NP) were the active catalysts, they were mostly prepared by reduction of supported Ru^3+^ salts [34,35,36].

The subject of the present work is the introduction of metal carbonyl cluster (MCC) precursors in the preparation of catalysts based on supported ruthenium nanoparticles (NP). The prepared catalysts have been tested in aerobic oxidation of HMF to FDCA in aqueous media. It has been shown that the FDCA formation in aqueous media is optimized using a dissolved base or by the use of a basic support for the catalyst. We have tested Ru NP from MCC in both conditions, by depositing Ru on a standard TiO_2_ support or by preparing basic oxide supports from LDH decomposition. The results have been compared to the ruthenium catalyst prepared by classical methods of salt impregnation.

## 2. Results and Discussion

### 2.1. Characterisation of the Catalyst Supports

**Figure 1:**
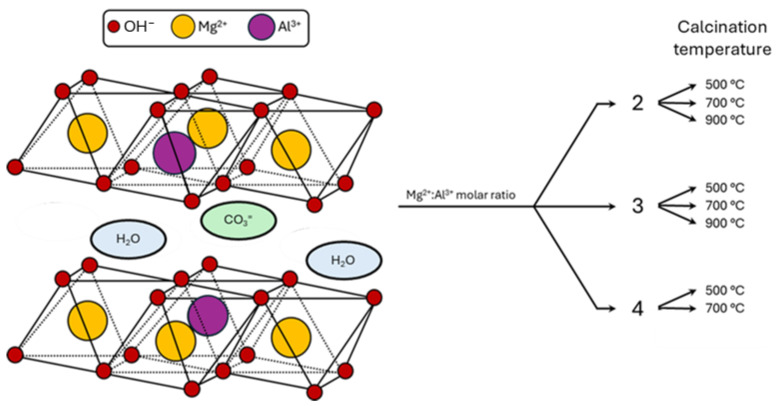
Representation of the prepared Mg/Al-based materials.

Titania is a classical support for metal nanoparticles in HMF oxidation catalysts [37,38,39,40,41]. The commercial DT51 TiO_2_ used in this project presented anatase NP with 20 nm average diameter and a surface area of 80 m^2^ g^−1^ (Figure S1). The oxidation of HMF in aqueous media is usually activated using a soluble base, which acts both as a product scavenger and a cocatalyst [42]. The deposition of metal nanoparticles on supports with basic functionalities has allowed to avoid the use of basic solutions. Al-doped MgO, called here Mg(Al)O, has been shown as an effective basic support for the oxidation of HMF to FDCA [43,44,45]. Mg(Al)O with different compositions have been prepared by thermal decomposition of hydrotalcite layered double hydroxides (LDH) with the general formula Mg_2x_Al_2_(CO_3_)(OH)_4(x+1)_(H_2_O)_y_, where x is the Mg/Al ratio. Trivalent aluminium is in a substitutional position for divalent magnesium in brucite-like M(OH)_2_ layers, generating a positive charge that is compensated by hydrated carbonate anions in the interlayer space. Samples have been prepared with Mg/Al ratio x = 2, 3, or 4, and named Mg2Al, Mg3Al, and Mg4Al, respectively, based on their Mg/Al ratio. Three batches of each precursor LDH have been calcined at 500, 700, and 900 °C (Figure 1).

**Figure 2:**
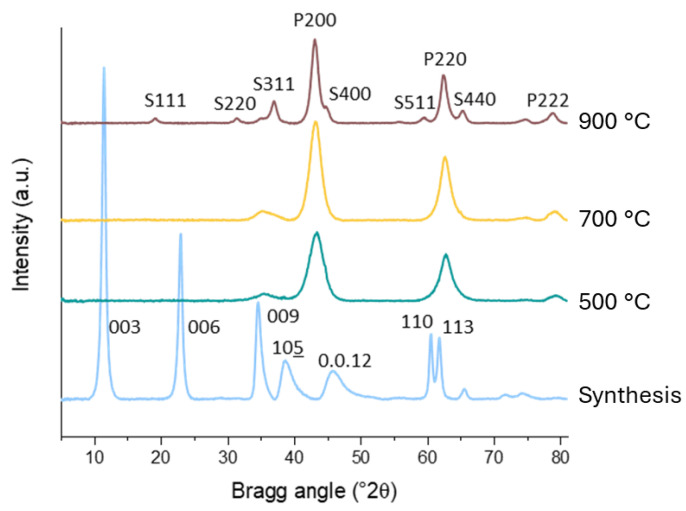
XRD patterns of the prepared LDH Mg3Al, with Mg:Al molar ratio of 3, calcined at different temperatures (from above 900, 700, 500, and 120 °C). Miller indexes hkl are given for the LDH structure in the as-synthesized sample and for the periclase (Phkl) and spinel (Shkl) phases in the calcined samples. Underlining is the normal shorthand for the negative Miller index.

The XRD patterns of as-synthesized Mg3Al LDH and its products of thermal activation at 500, 700, and 900 °C are reported in Figure 2. Mg(Al)O samples with different Mg/Al ratio present similar evolutions, as shown in Figure S2 in the Supplementary Information. The as-synthesized precursors present the XRD pattern of well-crystallized LDHs. The cell parameters, reported in Table 1, show a cell shrinkage at increasing Al content (lower Mg/Al ratio). This is due, in the case of a parameter, to the decrease of the average ionic radius, by replacement of a fraction of the Mg^2+^ cations (ionic radius 0.72 Å) [46] by the smaller Al^3+^ (0.53 Å). The shrinkage of the c parameter with Al substitution is attributed to a stronger electrostatic interaction of the charged layers with the interlayer carbonate anions [47].

The thermal treatment of the LDH precursors induces dehydration below 200 °C and deanionization and dehydroxylation between 200 and 350 °C (see TG curves in Figure S3), forming an amorphous material from which an Al-doped periclase MgO phase crystallizes upon calcination. The XRD pattern after calcination at 500 °C shows diffraction peaks (200, 220, and 222 [48]) of the periclase structure with cell size a = 4.177 Å, as reported in Table 1. This value, lower than the value 4.211 Å expected for pure MgO, indicates that the material is a Mg(Al)O solid solution with smaller Al^3+^ cation replacing a fraction of Mg^2+^ cations. It can be observed that the cell parameter does not strictly follow a Vegard’s law proportionality with the average ionic radius, as the insertion of a trivalent Al^3+^ does not only modify the average M-O distance but induces cation vacancies, which also affect the cell size [49]. The cation vacancies induced by partial replacement of divalent Mg^2+^ by trivalent Al^3+^ in the rock-salt structure are important for the reactivity of the material as they contribute to the stronger basicity of Mg(Al)O compared to pure MgO, through the formation of poorly coordinated oxygen and the decrease of the electronegativity of the material [50]. However, establishing a correlation between the cell parameter of Mg(Al)O and its Al content is especially tricky, as it is difficult to assess and evaluate the presence of XRD-undetected amorphous alumina, which can decrease the fraction of Al in the rock-salt structure. This effect has heavily affected the early literature Vegard’s correlations proposed for the cell size of Mg(Al)O [51,52]. On the basis of the average ionic radius, an experimental cell size 4.177 Å has been attributed to an Al/(Mg + Al) fraction of 32%, under the assumption that all Al^3+^ of the material isomorphously substitutes Mg^2+^ in the Mg(Al)O phase. Such a value would be higher that the whole Al content of our Mg3Al and Mg4Al samples. More recent Vegard’s correlations for the Al-induced cell shrinkage of doped MgO have been established for solid solutions prepared by reactive sputtering [49]. According to these evaluations, a cell size 4.177 Å would correspond to an Al^3+^ content of nearly 5%, allowing for the presence of a significant amount of amorphous alumina in our samples. The presence of α-Al_2_O_3_ in the calcination products of MgAl LDH at 850 °C has been confirmed by electron diffraction methods [53]. The incertitude on the Al content of the rock-salt structure is further increased by the presence, in the XRD patterns of all our calcined samples, of a small hump at 35.4° 2θ (2.54 Å), often observed in the calcination products of LDH and attributed to the 311 reflection of a highly defective spinel phase with poorly-defined Mg/Al ratio [54,55]. The low occupancy of the cation sites would justify the 8.38–8.41 Å cell parameter of this phase, much larger than the 8.083 Å cell of a stoichiometric MgAl_2_O_4_ [56,57].

Mg(Al)O phases with larger unit cell are formed when the LDH precursor is calcined at a higher temperature (see Table 1). The parameter approaches the value expected for pure MgO, indicating that less Al is incorporated in the rock-salt solid solution at high temperature. After calcination at 900 °C, an aluminium-richer spinel phase appears, with cell parameter a = 8.02–8.03 Å, significantly smaller than the value 8.083 typical of a direct MgAl_2_O_4_ spinel [58]. A cell size smaller than direct MgAl_2_O_4_ is generally observed in spinels formed at high temperatures, as thermal disorder induces a degree of inversion, with partial occupation of tetrahedral sites by Al^3+^ and corresponding occupation of the octahedral sites by Mg^2+^ [59,60]. The amount of spinels formed changes with the Mg/Al ratio of the parent material. The weight fraction of spinel, as evaluated by Rietveld analysis of the crystalline phases, increases from 12% (*w*/*w*) in Mg-rich Mg4Al to 27% in Al-rich Mg2Al. These values are roughly proportional to the Al/(Al + Mg) atomic fraction in the material, 20% in Mg4Al and 33% in Mg2Al, suggesting that the availability of aluminium is the limiting factor for the formation of the spinel phase at 900 °C.

The surface area from BET analysis and the average crystallite size calculated by the Scherrer formula are reported in Table 2 for the materials calcined at different temperatures. The crystallite size of the Mg(Al)O solid solutions increases with the temperature of calcination from 3.8–5 nm at 500 °C to nearly 8 nm at 900 °C. The size of spinel nanocrystals formed at 900 °C is in the 9–10 nm range. The surface area, between 91 and 115 m^2^ g^−1^ for the materials calcined at 500 °C, increases to 141–195 m^2^ g^−1^ for the materials calcined at 700 °C and decreases again to 101–122 m^2^ g^−1^ after calcination at 900 °C. The observed maximum of the surface area corresponds to a temperature much higher than the decomposition of the LDHs, completed at about 350 °C. It has been suggested that the dehydroxylation–deanionization of the parent material generates an occluded microporosity that becomes accessible to gas adsorption only by the aggregation of the oxide crystallites with the increase of temperature [61]. Further growth and sintering of the crystallites at higher temperatures induce the decrease of surface area beyond 700 °C.

**Figure 3:**
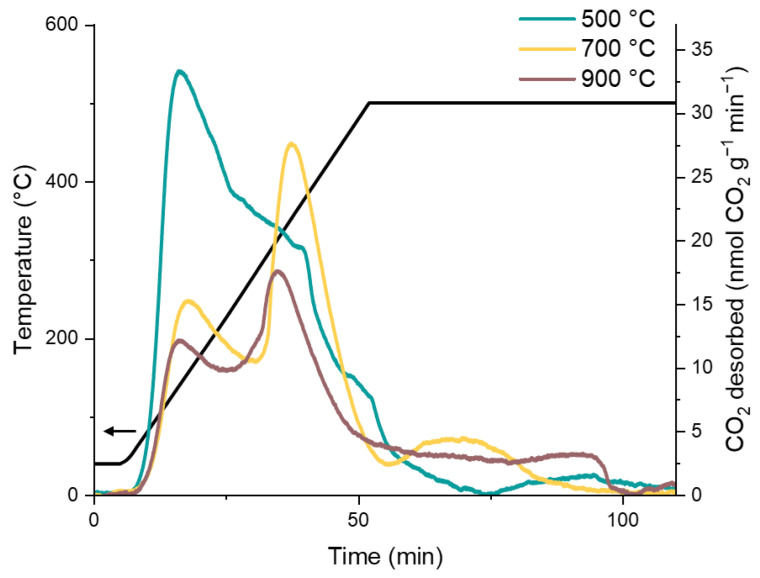
CO_2_-TPD traces of Mg3Al calcined at different temperature levels. The black line indicates the temperature ramp.

The temperature of desorption of adsorbed CO_2_ provides information on the number and strength of the basic sites of the material (Figure S4). The evolution of the CO_2_-TPD (temperature-programmed desorption) traces with the calcination temperature can be followed in Figure 3 in the case of sample Mg3Al. A first well-defined peak is observed at 145–165 °C, with an intensity that rapidly decreases with the calcination temperature. The sample calcined at 500 °C presents a complex pattern of desorption between 250 and 500 °C. After calcination at 700 °C, this pattern is simplified into a well-defined peak at 350 °C, which decreases in intensity with calcination at higher temperatures (Figure S4). For the samples calcined at the highest temperature levels, the desorption of CO_2_ continues at the constant temperature of 500 °C for 30–40 min. This kinetically delayed desorption is indicative of transport limitations in the material, likely induced by the growth and sintering of the crystallites at high temperatures.

Basic sites are usually classified as weak, medium, and strong at increasing temperatures of CO_2_ desorption [62]. The effective temperature of desorption corresponding to each kind of site is extremely dependent on the experimental conditions of the TPD experiments [63,64]. The nature of the adsorption sites of CO_2_ on MgO and mixed Al-Mg oxides has been characterized in detail by FT-IR spectroscopy methods [65,66,67,68]. The first CO_2_ desorption peak corresponds to the decomposition of surface bicarbonate species formed by the interaction of CO_2_ with surface hydroxyls. Further CO_2_ is released at higher temperatures by the decomposition of surface carbonates. Monodentate carbonates, formed by the interaction of CO_2_ with electron donor surface oxygens, and bidentate carbonates, bridging surface oxygens and cations, can be observed. The two kinds of carbonate sites imply the same surface oxygens and can be reciprocally converted by changes of the degree of hydration of the surface. Temperature-induced dehydroxylation liberates coordinatively unsaturated cations and favors the formation of bidentate carbonates. The shift of the broad desorption complex between 250 and 500 °C in Figure 3 towards a well-defined peak probably corresponds to the conversion of monodentate to bidentate carbonates with the dehydration of the surface at the increase of the calcination temperature.

The total amount of desorbed CO_2_ is reported for each sample in Table 3. The fraction of CO_2_ released by surface hydroxyls can be evaluated by deconvolution of the initial peak (Figure S4) and is reported in Table 3 as weak basic sites (w.b.s.). Further CO_2_ release up to 500 °C corresponds to medium and strong basic sites (m.s.b.s.), and CO_2_ released in the isothermal treatment at 500 °C is labelled as poorly accessible basic sites (p.a.b.s.). Despite some differences between samples, the data indicate a common trend of evolution of the basicity with the calcination temperature. The rise of the calcination temperature from 500 to 700 °C induces a severe decrease in the amount of adsorbed CO_2_, which shifts from 2.1–3.2 to 0.8–1.3 μmol m^−2^. This effect is generally accompanied by a decrease of the w.b.s., corresponding to a dehydroxylation of the surface with the increase of the calcination temperature. A partial exception is represented by Mg2Al, which retains a high w.b.s. fraction despite the increase in calcination temperature. This behaviour can tentatively be related to the high content of trivalent Al^3+^, which induces cation vacancies with poorly coordinated oxygen and reduces the electronegativity of the material.

The calcination at the higher temperature of 900 °C appears to increase again the amount of desorbed CO_2_ by unit of surface. However, the amount of total CO_2_ per mass unit is nearly constant between calcination at 700 and 900 °C. Indeed, the increase of CO_2_ amount by unit of surface is induced by a decrease in surface area with calcination. It is worth observing that the rise of CO_2_ amount per surface unit corresponds to an increased contribution of p.a.b.s., i.e., CO_2_ kinetically retained at 500 °C. It seems likely that the p.a.b.s. had to be related to the diffusion of CO_2_ from bulk carbonate sites, which are constrained by the sintering of the material and do not correspond to a surface area measurable by N_2_ sorption.

### 2.2. Characterization of the Ruthenium Catalysts

**Figure 4:**
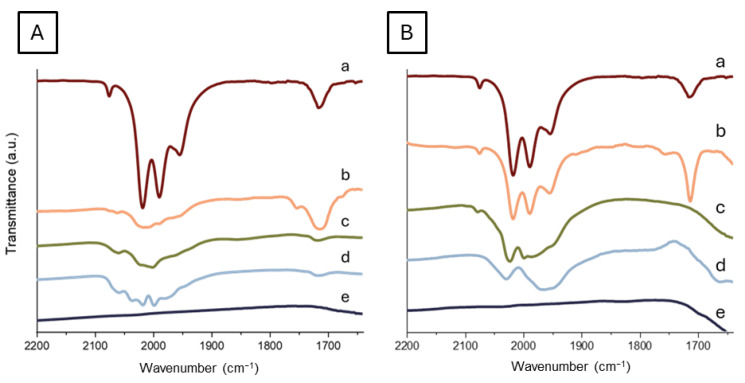
FTIR spectra recorded during the [HRu_3_(CO)_11_]^−^ impregnation protocol on TiO_2_ (**A**) and Mg(Al)O material with a molar ratio Mg/Al 3 calcined at 700 °C (**B**). Each spectrum was recorded during different steps of the preparation: cluster dissolved in CH_3_CN (a), cluster and support suspension in CH_3_CN (b), dried catalyst powder (c), catalyst powder after thermal treatment at 120 °C under N_2_ (d), and catalyst powder after a second thermal treatment at 350 °C for 2 h under reductive (H_2_/N_2_ 10% *v*/*v*) atmosphere (e).

Catalysts with 1.5% Ru load (*w*/*w*) were prepared by deposition of a metal carbonyl cluster on TiO_2_ and Mg(Al)O supports, and the results were compared with a bench catalyst prepared by RuCl_3_ impregnation (Table S1). The cluster used for the preparation of Ru NP was [HRu_3_(CO)_11_]^−^, itself an effective water–gas shift homogeneous catalyst [69,70]. The decomposition of the parent carbonyl cluster on the support is a major step in the formation of the metal nanoparticles, which can be followed by the evolution of the νCO bands in the carbonyl region of the IR spectra during the main steps of the catalysts’ preparation procedure (Figures S5–S7), reported in Figure 4 for the TiO_2_ and the Mg3Al support calcined at 700 °C.

**Figure 5:**
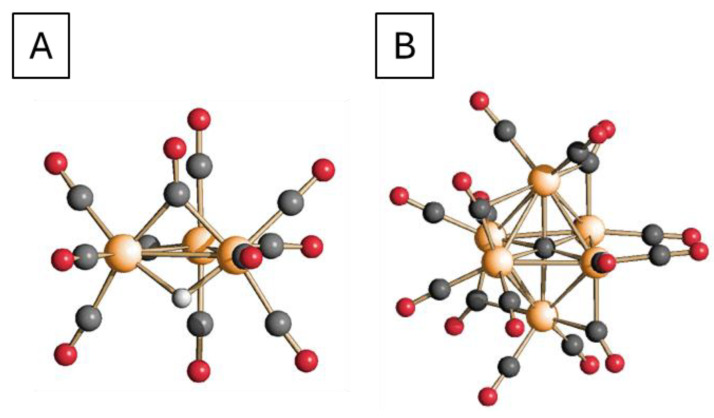
Molecular structure of [HRu_3_(CO)_11_]^−^ (**A**) and [Ru_6_C(CO)_16_]^2−^ (**B**) (orange, Ru; red, O; grey, C) represented based on literature data [73,74].

The clusters in solution (Figure 4A(a),B(a)) show the typical IR spectrum of the planar trigonal [HRu_3_(CO)_11_]^−^ (Figure 5A) cluster [71]. After the addition of the support, the behavior of the cluster on TiO_2_ and Mg(Al)O differs, due to a different interaction with the surface. In the case of the TiO_2_ support, as soon as titania is added, the aspect of the IR spectrum changes, indicating an interaction between the support and the cluster (Figure 4A(b)). The observed shift suggests the formation of the high nuclearity cluster with the formula [Ru_6_C(CO)_16_]^2−^ (Figure 5B), and the process is completed after the first thermal treatment (Figure 4A(c)). In agreement with the formation of the carbide cluster, a weak band in the edge-bridging CO region is observed in the spectra c and d of Figure 4A, which confirms the presence of the highly symmetrical octahedral carbidocarbonyl [Ru_6_C(CO)_16_]^2−^ cluster [72].

In the case of Mg(Al)O, no significant reaction is observed upon the addition of the support to the cluster solution before removal of the solvent (Figure 4B(b)). After drying in vacuo, the starting cluster seems to be still present even though the disappearance of νCO bands in the μ-CO region and the broadening of the terminal νCO bands indicate an interaction between the cluster and the support (Figure 4B(c)). This might be explained by partial substitution of some CO ligand in the Ru3 cluster with Mg-bonded oxygen atoms and the formation of species such as Ru_3_(CO)_6_(μ-OMg)_3_ [75]. This process is completed after thermal treatment at 120 °C (Figure 4B(d)), suggesting a higher stability of the trigonal ruthenium structure but confirming that strong interaction with the surface of the basic oxide induces partial decarbonylation of the cluster. Similar carbonyl decomposition patterns, as observed for different temperatures of calcination and on other Mg(Al)O supports, are reported in the Supplementary Information.

On both TiO_2_ and Mg(Al)O supports, the thermal treatment at 350 °C in a reducing atmosphere brought the complete disappearance of the carbonyl-related bands, with the formation of metallic ruthenium nanoparticles. However, the different patterns of cluster decomposition on TiO_2_ and Mg(Al)O indicate a different interaction with the support. It appears that, upon desolvation and heating, [HRu_3_(CO)_11_]^−^ tends to rearrange to Ru6 clusters upon TiO_2_ whereas, on Mg(Al)O, it establishes stronger bonds with the surface oxygens retaining Ru3 structure.

**Figure 6:**
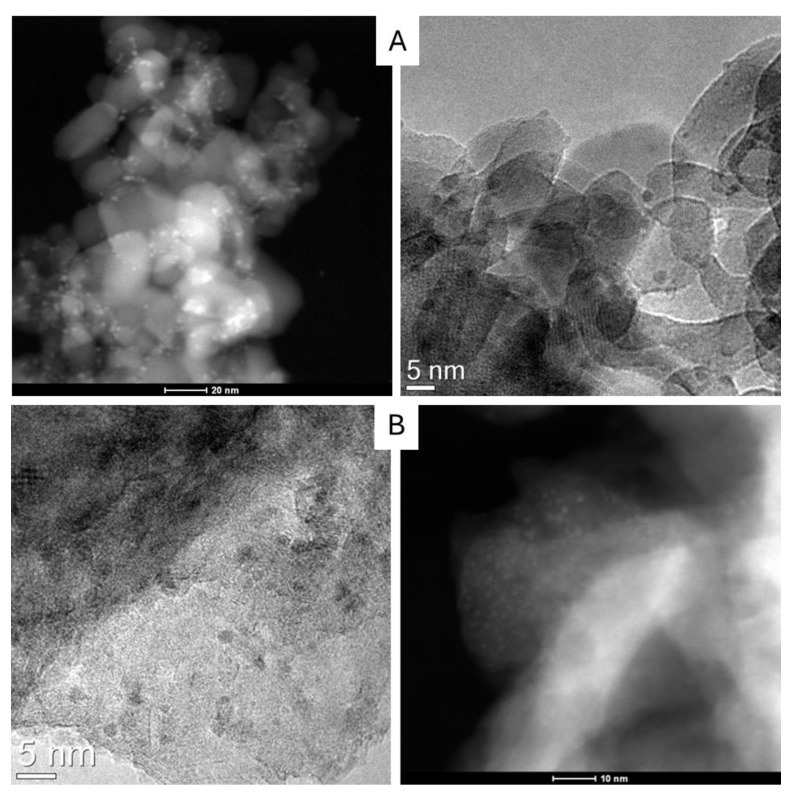
TEM/HAADF images collected on the two catalysts Ru/TiO_2_ (**A**) and Ru/Mg(Al)o prepared with the Mg/Al-based support with a Mg:Al molar ratio of 3 and calcined at 500 °C (**B**). MCCs were used as NPs precursors. Nanoparticles size distributions are reported in Figure S9.

The surface area analysis conducted on both the materials before and after the impregnation did not show relevant changes, suggesting the formation of small nanoparticles that do not block the porosity of the supports (Table S2). Transmission electron microscopy (TEM) allows identification of the metal nanoparticles formed on the supports, both by carbonyl decomposition and by a standard RuCl_3_ impregnation method (Figure 6, Figures S8 and S9). The average size of metal nanoparticles reported in Table 4 decreases with the method of preparation from 1.8 to 1.2 nm in the order RuCl_3_/TiO_2_ > cluster/TiO_2_ > cluster/Mg(Al)O. In the case of the RuCl_3_ preparation, larger agglomerates of RuO_x_ were observed (Figure S8). The obtention of smaller particles on the most basic support is in good agreement with the prevention of metal sintering expected with the increase of electron donor activity of the support [76].

### 2.3. Preliminary Catalytic Tests

**Figure 7:**
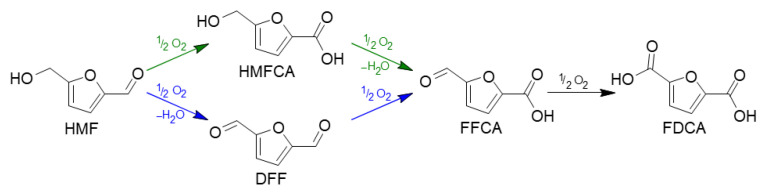
Reaction network for the HMF oxidation to FDCA.

All the prepared catalysts were identified as Ru/XX-YYY-C/S with XX being the support, YYY the calcination temperature of Mg/Al-based material and C or S for cluster or salt derived-systems. The samples were tested in the HMF oxidation to produce FDCA (Figure 7), performing the catalytic tests in a batch reactor employing water as a solvent and charging a specific oxygen pressure before starting the heating ramp to carry out the reactions at the targeted temperature. The HMF oxidation occurs through several steps due to the two different functional groups in the furanic ring. The first step involves the oxidation of one of the two groups of the molecule, the aldehyde or the alcoholic group. The oxidation of the former leads to the formation of 5-hydroxymethyl-2-furancarboxylic acid (HMFCA), while the oxidation of the latter produces 2,5-diformylfuran (DFF). Then, the consecutive oxidation of both these compounds leads to the formation of 5-formyl-2-furancarboxylic acid (FFCA), and, finally, its consecutive oxidation allows the production of 2,5-furandicarboxylic acid (FDCA) [77,78].

Before starting the evaluation of the activity of each prepared catalyst, some blank tests were carried out on the reaction solution both in the absence of any heterogeneous catalyst and using only the desired supports without the active phase of metal NPs. The results of the tests are reported in Table 5. The blank tests without the supports were conducted both using the solution of HMF dissolved in water alone and, with the addition of a homogeneous base (NaHCO_3_), necessary to promote the reaction when the Ru/TiO_2_ catalyst was used. The HMF solution alone resulted in a 4% conversion and no product could be identified, while the addition of Na_2_CO_3_ raised the conversion to 26% and a limited formation of HMFCA, FFCA, and FDCA was observed. The important by-product formation (identified as “Others” in the Table 5) was clearly due to side-reactions promoted by the presence of the base, with ring-opening of HMF to levulinic and formic acids and condensation to polymeric humins [37,79]. A similar effect of the base addition was observed in the blank tests on TiO_2_. When the support was tested in base-free conditions, a conversion of 8% to unidentified products was observed, whereas, in the presence of base, the reaction rose to 35% and some HMFCA, FFCA, and FDCA were observed together with major products to be the side-products.

The Mg(Al)O material with a Mg/Al molar ratio equal to 3 and calcined at the three targeted temperatures—500, 700, and 900 °C—was tested in the absence of base, and 26–28% conversion was measured. The yield of products of the reaction chain leading to FDCA was significantly affected by the temperature of calcination of the material, decreasing from 14 to 8% with the rise of calcination temperature from 500 to 900 °C. It can be observed that, on Mg(Al)O supports, more DFF than HMFCA was measured, at the difference of the results observed in the presence of a soluble base. These tests confirmed the necessity of the Ru nanoparticles to carry out selective oxidation.

### 2.4. Activity of Ru/TiO_2_

**Figure 8:**
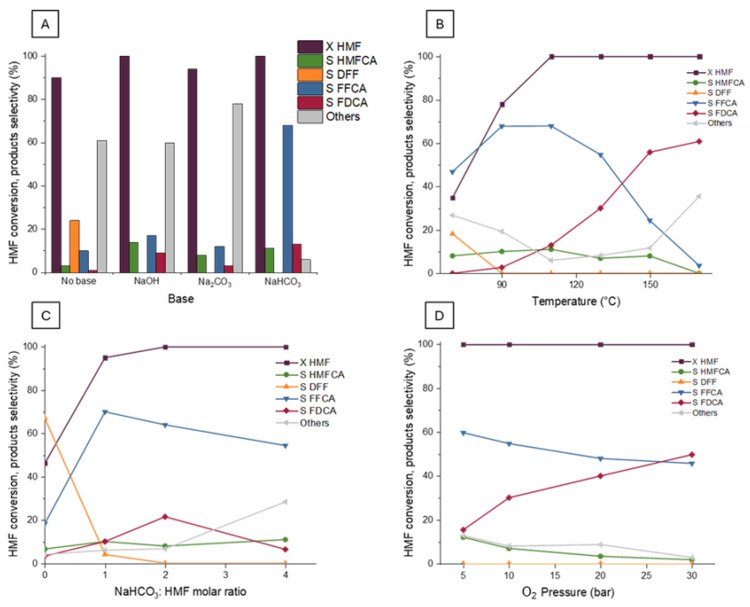
(**A**) Effect of the homogeneous base added to the reaction solution using Ru/TiO_2_ catalyst. Operative conditions: time 4 h, temperature 110 °C, O_2_ pressure 10 bar, molar ratio HMF/Ru = 100, molar ratio base/HMF = 2. HMF conversion and products selectivity as a function of molar ratio temperature (**B**), NaHCO_3_:HMF (**C**) and oxygen pressure (**D**) using Ru/TiO_2_ catalyst. Operative conditions: time 2 h (**C**) or 4h (**B**–**D**), temperature 130 °C (**C**,**D**), O_2_ pressure 10 bar (**B**,**C**), molar ratio HMF/Ru = 100, molar ratio NaHCO_3_/HMF = 2 (**B**–**D**). “Others” refers to unidentified side products.

Concerning the Ru-based catalysts prepared with TiO_2_ as support, the use of a homogeneous base was necessary to reach FDCA production due to the lack of the support basic properties [80]. For this reason, a first study on the use of different homogeneous bases was carried out employing the Ru/TiO_2_-C catalyst. This study was conducted using experimental conditions already used in previous works on HMF oxidation (110 °C, 4 h, 10 bar of O_2_), maintaining an HMF:Ru molar ratio of 100 (optimized experimental conditions) and adding a specific amount of a chosen homogeneous base to the reaction mixture to achieve a final molar ratio of base/HMF = 2. The selected homogeneous bases for this study were NaOH, Na_2_CO_3_, and NaHCO_3_, and the results obtained from the catalytic tests were compared to those of a test conducted without employing a homogeneous base (Figure 8A). Carrying out the reaction using the Ru/TiO_2_-C catalyst without employing a base led to a conversion of HMF around 90%. Still, only a low selectivity in the intermediate products was obtained (20% for DFF and less than 10% for FFCA), and most of the reagent was converted into by-products. Using stronger homogeneous bases, such as NaOH and Na_2_CO_3_, also resulted in a high quantity of by-products, such as ring-opening and condensation products of the HMF degradation, and low selectivities (lower than 20%) in the reaction intermediates. However, using NaHCO_3_ as the homogeneous base had a positive effect in terms of activity and especially selectivity to the desired products. In fact, a complete HMF conversion and selectivity in FFCA higher than 70% and FDCA close to 10% were reached after 4 h of reaction. It appears that strong alkaline conditions (Na_2_CO_3_ and NaOH led to a pH in the reaction media around 11 and 12) can lead to degradation reactions instead of promoting FDCA production [80]. For this reason, NaHCO_3_ was chosen as the optimal base for conducting the catalytic tests, since its employment leads to a pH of the reaction media around 8.3, reducing the degradation reactions.

A study on the stirring rate was performed to confirm that no transfer limitation affected the results of the catalytic tests. In particular, the stirring rate was changed between 200 and 1200 rpm and four tests were carried out for 10 min at 110 °C employing a NaHCO_3_/HMF molar ratio of 2. The obtained results are reported in Figure S10, and it can be noted that, increasing the stirring rate from 200 to 400 rpm, the values of reagent conversion increased more than twice and remained stable when the stirring rate was further increased to 600 and 1200 rpm. These results suggested that no diffusional external limitations happened when the tests were conducted at stirring rates higher than 400 rpm, so the system used to conduct the catalytic tests allowed a proper reagent diffusion to the surface of the active sites. For this reason, all the tests shown in this work were conducted using a stirring rate of 1200 rpm (with 1500 rpm being the highest possible speed).

After these preliminary studies, the influence of the different reaction parameters was considered. In particular, the reaction temperature, the molar ratio NaHCO_3_/HMF, and the oxygen pressure were evaluated (Figure 8B,C). Several tests were carried out by changing the reaction temperature from 70 to 170 °C (Figure 8B), maintaining the oxygen pressure at 10 bar and a NaHCO_3_/HMF molar ratio of 2. The obtained results showed that the reagent conversion increased with the temperature, from values lower than 40% at 70 °C to complete conversion of HMF for temperature of 110 °C or higher. Concerning the first two reaction intermediates, DFF was observed only for low reaction temperature (at 70 °C), while HMFCA was always detectable, with a selectivity of around 10%, and only at 170 °C its selectivity dropped to 0. The decrease of DFF at the increase of FFCA suggests DFF as the major intermediate towards successive oxidation products. Further investigation into the reaction mechanism will be presented later. Concerning the FDCA selectivity, it can be noted that a rapid increase happened between 110 and 150 °C, but an improvement lower than 5% is observed between 150 and 170 °C. This effect has to be related to the evolution of the formation of unidentified products. At 70 °C, the selectivity in side-products was similar to the one observed in the blank test, mainly due to the reactivity of the soluble base. At higher temperatures, the side-product selectivity decreases to less than 10%, due to the competition with catalyzed pathways with higher activation energy. Above 150 °C, new thermally activated mechanisms of degradation increase the selectivity of coproducts towards 40%, at the expense of FDCA and HMFCA. Analyzing the results obtained from this study, the reaction temperature selected to carry out successive tests was 130 °C. At this temperature, the catalyst showed good activity, allowing the complete conversion of the reagent and the formation of products with interesting selectivity (FFCA around 50% and FDCA around 30%). Moreover, a low amount of by-products was formed (lower than 10%). These results suggested that at this temperature there was a good compromise between the catalytic performances, allowing better assessment of the differences among the catalysts.

Since the employment of Ru as the active phase requires the presence of a weak homogeneous base such as NaHCO_3_ to promote the HMF oxidation to FDCA, an evaluation of the molar ratio base/reagent was necessary. For this reason, some tests were carried out varying the NaHCO_3_/HMF molar ratio between 0 and 4 (Figure 8C), highlighting the necessity of this kind of reaction promotion. Indeed, the absence of the base only allowed HMF conversion lower than 50%, while adding one equivalent of the base led almost to a complete conversion. Concerning the evolution of the selectivities varying the amount of NHCO_3_, it can be noted that the FFCA selectivity decreased with the increase of the base amount, while FDCA reached its highest selectivity for a molar ratio of 2. Moreover, the amount of by-products increased with the amount of the base, exceeding 20% when the base:HMF molar ratio was 4. From this evaluation, using 2 base equivalents with respect to the HMF proved to be a good option in terms of catalytic performance and selectivity to the desired products. The last parameter studied was the O_2_ pressure charged before starting the reaction (Figure 8D). In particular, these tests were conducted by varying the oxygen pressure from 5 to 30 bar. The HMF conversion reached 100% in each test. However, the selectivity of the different products varied. The selectivity of FFCA slightly decreased when the O_2_ pressure increased, while the FDCA selectivity increased, as the reaction further proceeded. In all the pressure ranges considered, the amount of by-product remained low and relatively stable, being less than 10%. Despite the increase in FDCA selectivity between 10 and 30 bar, it was decided to conduct the tests in milder conditions, maintaining the pressure at 10 bar, to better observe differences between the tested catalysts.

### 2.5. Comparison Between TiO_2_- and Mg(Al)O-Based Ruthenium Catalysts

**Figure 9:**
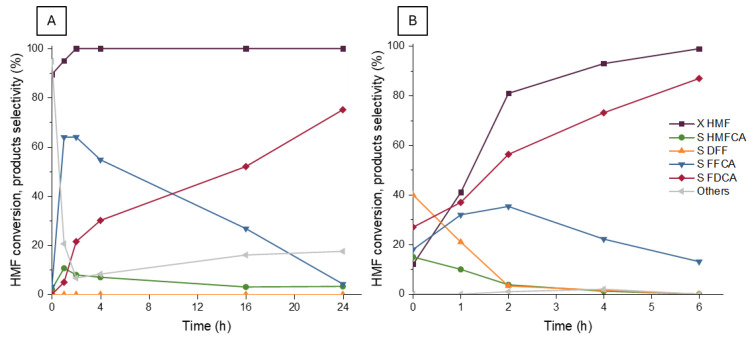
HMF conversion and products selectivity as a function of time using Ru/TiO_2_-C (**A**) and Ru/Mg(Al)O-3-500 (**B**) catalyst. Operative conditions: temperature 130 °C, O_2_ pressure 10 bar, molar ratio HMF/Ru = 100. The catalyst Ru/TiO_2_ was tested with a homogeneous base: molar ratio NaHCO_3_/HMF = 2.

Using the optimized reaction conditions (130 °C and 10 bar of O_2_ pressure), tests at different reaction times were carried out using the catalysts Ru/TiO_2_-C with 2 equivalents of NaHCO_3_ and Ru/Mg3A-500-C with no homogeneous base. The results obtained from these experiments (Figure 9) highlighted some interesting differences in the progression of the reaction using the two catalysts.

Focusing on the results of the Ru/TiO_2_-C catalyst (Figure 9A), a complete conversion of HMF occurred after just two hours of reaction, increasing from 90% to 95% after the first reaction hour. The DFF was never detectable, while the HMFCA selectivity was stable around 5/10% in all the range of reaction time considered. The low level of the initial reaction intermediates, DFF and HMFCA, did not provide information on their relative role in the formation of FFCA. These two intermediates were used in test reactions as starting reagents instead of HMF, in the same reaction conditions, to better understand which one was more favored as an oxidation product of HMF. The results, reported in Table 6, indicate that DFF was completely converted after 4 h of reaction, while HMFCA conversion only reached 39%. Considering also the evolution of these two compounds as a function of time, it can be confirmed that the reaction kinetics favored the reaction pathway that involves DFF formation as the first reaction intermediate. Probably, both intermediates were formed from the HMF oxidation, but the DFF conversion to FFCA was faster than that to HMFCA, explaining the absence of the former intermediate and the stable presence of the second in all tests.

Considering the evolution of reaction products, in Figure 9A, of the selectivity of consecutive products, FFCA and FDCA, it can be noted that after two hours, the FFCA selectivity reached its maximum of almost 65% and began to decrease, while the selectivity towards FDCA increased from 20% after 2 h, reaching almost 80% after 24 h of reaction. The oxidation of FFCA to FDCA slowly proceeded with the progress of reaction time, and the low conversion observed (around 25%) of FFCA when it was used as a reagent (Table 6) suggested that this last oxidation represents the rate-determining step of the reaction.

On the other hand, employing support with basic properties (Figure 9B) instead of the homogeneous base to promote the reaction led to a complete conversion of the substrate after 6 h of reaction, instead of 2 h on TiO_2_ with added base. However, a noteworthy FDCA selectivity of around 85% was reached after 6 h, higher than the selectivity obtained on TiO_2_ after 24 h. At low reaction time, significant selectivity in DFF and HMFCA was observed. Both decreased to values below 5% after the first two hours of reaction, while the FFCA selectivity reached a maximum (more than 30%) at 2 h, after having decreased due to its oxidation to FDCA. In the case of TiO_2_, the maximum of FFCA was reached at the same time but with a selectivity higher than 60%. The differences in the products’ evolution with time highlighted a different behavior of the ruthenium catalyst on different supports. The initial oxidation of HMF and FFCA formation occurred faster when a homogeneous base and TiO_2_ were employed. However, the oxidation of FFCA to FDCA was much faster when support with basic properties was employed. It can also be observed that the kinetic advantage of the DFF pathway on HMFCA in the FFCA formation was less marked on the basic support than in the presence of a soluble base.

### 2.6. Effect of Mg/Al Ratio and Calcination Temperature on the Activity of Ru/Mg(Al)O Catalysts

**Figure 10:**
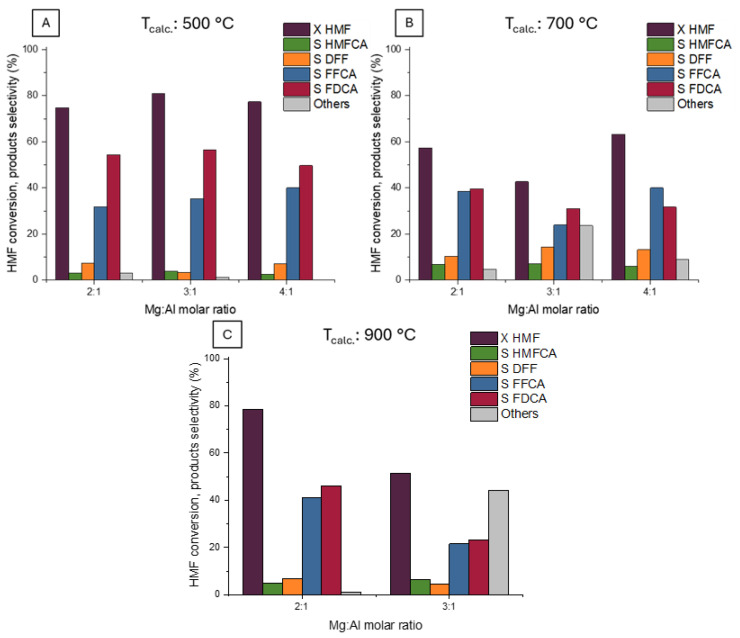
Effect of the catalytic support. The graphs compare the conversion and Mg/Al molar ratio of the materials calcined at the same temperature: 500 °C (**A**), 700 °C (**B**), and 900 °C (**C**). Operative conditions: time 2 h, temperature 130 °C, O_2_ pressure 10 bar, molar ratio HMF/metal = 100.

The positive effect of the basic support on the formation rate of FDCA justified a fine-tuning of the support properties. Tests were carried out by preparing three different LDH-type materials, employing different Mg:Al molar ratios (2, 3, and 4) and calcining all of them at different temperatures (500, 700, and 900 °C). The final Mg(Al)O materials were used as supports for Ru nanoparticles prepared through cluster impregnation and decomposition. All the final catalysts were tested in the base-free HMF oxidation (Figure 10), using the optimized reaction conditions for the Ru/TiO_2_ catalyst (130 °C and 10 bar of oxygen pressure) and choosing a short reaction time of 2 h to better highlight the differences between the catalysts.

The XRD and CO_2_-TPD results showed a common trend of evolution of the three materials with the temperature of calcination, as well as some finer differences as a function of the Mg/Al ratio. The materials with all Mg/Al ratios calcined at 500 °C shared similar surface area, phase composition and basicity. In the same way, the catalytic activity was very similar for the three catalysts calcined at the same temperature, as presented in Figure 10A, with conversion 75–80%, selectivity of FDCA 50–57%, and FFCA 33–40% observed. The high ratio of FDCA/FFCA for all catalysts confirms the interest in using a basic support for the limiting step of the FDCA synthesis.

**Figure 11:**
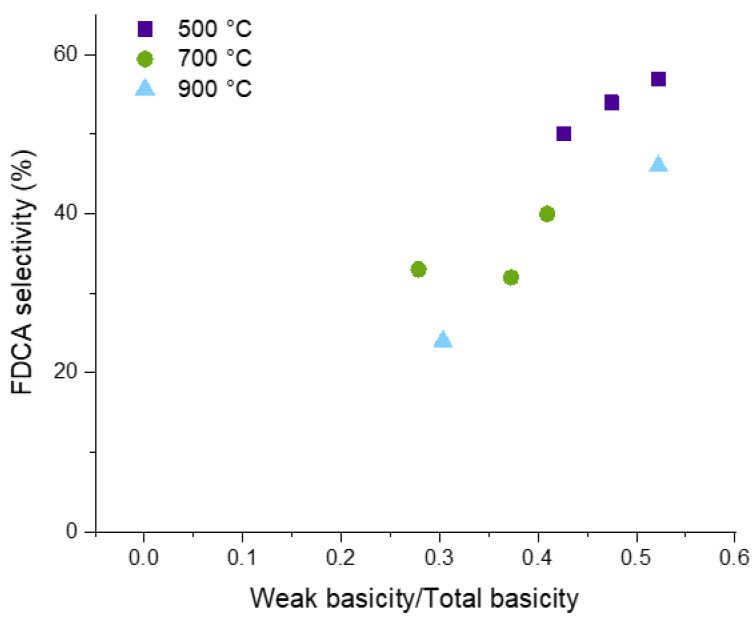
Correlation between the fraction of weak basic sites and the FDCA selectivity obtained on the Ru-based systems supported on the Mg/Al materials.

Calcination at higher temperatures led to lower conversion on all catalysts, with values of 57, 43, and 61% for Mg2Al, Mg3Al, and Mg4Al, respectively, calcined at 700 °C. This decrease can be well correlated to the large decrease of total basicity for all materials (see Table 3). Calcination at 900 °C improved the recovery of the conversion for supports calcined at 900 °C. In this case, conversions of 79, 52, and 56% of HMF were observed on Mg2Al, Mg3Al, and Mg4Al, respectively. A similar trend was also observed for the FDCA selectivity, which decreased to 40, 32, and 33% for Mg2Al, Mg3Al, and Mg4Al, respectively, after calcination at 700 °C and recovered for Mg2Al and Mg4Al at 47 and 37%, respectively, after calcination at 900 °C. The formation of unidentified side-products was roughly inversely proportional to the selectivity of FDCA. Side-products were especially relevant on the Mg-rich supports, reaching 24 and 44% on Mg3Al calcined at 700 and 900 °C, respectively. Different trends with the calcination temperature could also be observed on the intermediate of HMF oxidation to FFCA. For all samples, the selectivity of DFF reached a maximum of 10–15% for calcination at 700 °C, and the HMFCA selectivity slightly increased with the temperature of calcination up to 5–7% for calcination at 900 °C. This suggests that the kinetics of the two paths of formation of FFCA are affected in a different way by the thermal activation of the support. Probably the most interesting result of the Ru-Mg(Al)O catalyst is the high selectivity of FDCA. Taking into account the characterization of the catalyst, a correlation can be observed between the value of selectivity to FDCA and the fraction of weak basic sites in CO_2_-TPD data. Weak basic sites can be related to the amount of surface hydroxyls at the support surface, which is decreased by the calcination treatment at increased temperature (Figure 11).

The presence and amount of weak basic sites can affect the catalytic activity in different ways. It has been shown that the surface hydroxyls, corresponding to weak basic sites, can promote the activity of Mg^2+^-O^2−^ pairs, traditionally interpreted as medium basic sites, in basic catalysis. As it was in the case with the employment of a weak homogeneous base when the Ru/TiO_2_ catalyst was used, a more significant number of weak basic sites can enhance the reaction, whereas the presence of stronger basic sites tends to reduce the catalyst selectivity, leading to increased degradation of the reagent. This degradation is evident from the higher amounts of byproducts lowering the FDCA selectivity, as illustrated in Figure 11. The hydroxylation of the surface can also affect the formation of the metal nanoparticles. It was observed that a higher degree of hydroxylation of the surface favored the dispersion of metallic ruthenium at the surface of silica [81,82].

### 2.7. Comparison of Cluster- and Salt-Derived Ru Catalysts

**Figure 12:**
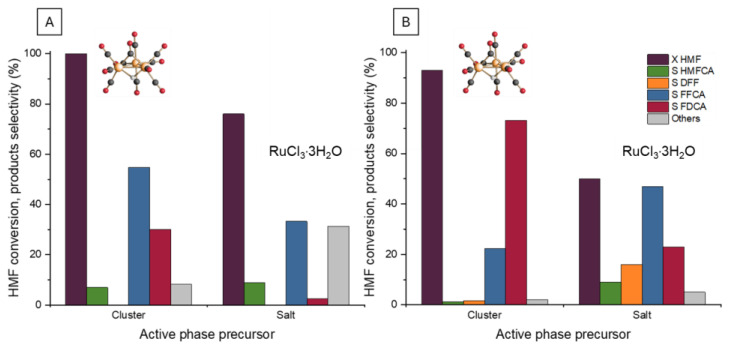
Comparison of Ru/TiO_2_ (**A**), and Ru/Mg(Al)O-3-500 (**B**) samples prepared by cluster decomposition or salt impregnation (RuCl_3_·3H_2_O). Operative conditions: time 4 h, temperature 130 °C, O_2_ pressure 10 bar, molar ratio HMF/total metal = 100. The catalyst Ru/TiO_2_ was tested with a homogeneous base: molar ratio NaHCO_3_/HMF = 2. (Concerning the cluster structure: orange, Ru; red, O; grey, C).

It is worth comparing the catalytic activity of the supported ruthenium nanoparticles prepared in this work through the decomposition of a metal carbonyl cluster with the anionic formula [HRu_3_(CO)_11_]^−^ with the activity of more classical Ru-based systems for the HMF oxidation, prepared through metal salt impregnation. The preparation of NPs through cluster decomposition usually leads to the formation of smaller nanoparticles well dispersed over the selected support, and significant nanoparticles-support interactions can be developed. In order to validate the methodology employed in this work, Ru nanoparticles-based catalysts were also prepared using RuCl_3_·3H_2_O for the active phase preparation on TiO_2_ and the Mg3Al-based material calcined at 500 °C as supports. These two catalysts were tested in the optimized reaction conditions (130 °C, 10 bar of O_2_ pressure and the Ru/TiO_2_-S with a NaHCO_3_/HMF molar ratio of 2) and the obtained results were compared to those obtained using the catalysts prepared with MCC as reported in Figure 12.

Both types of catalysts prepared employing metal carbonyl clusters showed enhanced activity. In particular, both the catalysts prepared with the cluster presented improved HMF conversion. Using the titania as support (Figure 12), the conversion on the cluster-derived catalyst was 100% and more than 20% higher than the catalyst prepared using RuCl_3_ as the desired precursor. On the Mg(Al)O support, the conversion increased more than 40% from the salt-prepared to the cluster-prepared NPs. Moreover, the selectivity in the more oxidized products, such as FFCA and FDCA, was lower on the systems prepared with the salts as precursors, together with higher quantities of by-products formed due to the lower activity of the catalysts, which does not prevent competitive HMF degradation processes from occurring. These differences in the catalyst activity can be related to the formation of smaller and more active nanoparticles when the clusters were used as NPS precursors, probably due to stronger interaction of these species with the support during the catalyst synthesis. Indeed, the average particle size of ruthenium was 1.8, 1.4, and 1.2 nm for Ru/TiO_2_ from salt, Ru/TiO_2_ from cluster, and Ru/Mg3Al from cluster, respectively (Table 6).

## 3. Materials and Methods

### 3.1. Materials

Magnesium nitrate hexahydrate (Mg(NO_3_)_2_·6H_2_O, 99.99%, Sigma Aldrich, Burlington, MA, USA), aluminium nitrate nonahydrate (Al(NO_3_)_3_·9H_2_O, 99%, Sigma Aldrich, Burlington, MA, USA), and sodium carbonate (Na_2_CO_3_, 99%, Sigma Aldrich, Burlington, MA, USA) were employed for the supports synthesis. Commercial reagents: Ruthenium(III) chloride trihydrate (RuCl_3_·3H_2_O), sodium methylate (NaOMe), tetraethylammonium bromide ([NEt_4_]Br) used for the ruthenium carbonyl cluster synthesis were purchased from Merck (Darmstadt, Germany). Commercial titania (TiO_2_, pure anatase phase, DT-51 Millennium Chemicals, now Tronox, Oklahoma City, OK, USA) was used as support for the catalyst’s preparation. For the catalytic tests, 5-hydroxymethylfurfural (AVA Biochem, Zug, Switzerland) and sodium bicarbonate (NaHCO_3_, >99.7%, Sigma Aldrich, Burlington, MA, USA) were used, and 2,5-diformylfuran, 5-hydroxymethyl-2-furancarboxylic acid, 5-formyl-2-furancarboxylic acid, and 2,5-furandicarboxylic acid (DFF, HMFCA, FFCA, FDCA) (Toronto Research Chemical, North York, ON, Canada and Sigma Aldrich, Burlington, MA, USA) were employed as reference commercial species for HPLC analysis.

### 3.2. Synthesis of Mg-Al Hydrotalcites with Different Mg:Al Molar Ratios

The synthesis of Mg-Al hydrotalcites was carried out by precipitation at controlled pH. Once the desired amount of layered double hydroxides (LDH) was determined, a 1 M solution of metal cation precursors (Mg(NO_3_)_2_·6H_2_O and Al(NO_3_)_3_·9H_2_O) and a solution of anion precursor (Na_2_CO_3_) were prepared, considering that its concentration should be twice that of trivalent cations (Al^3+^). The solution containing the base was placed under vigorous stirring and heated to a temperature between 50 and 60 °C. At this point, the solution containing the metal precursor was added dropwise to the Na_2_CO_3_ solution. The synthesis was conducted at a controlled pH of 10.5, measured by a pH meter, and maintained constant by dropwise additions of 3 M NaOH. Once all the precursor solution was added, the suspension was left in the digestion phase for 1 h, at the same temperature. The solid was then filtered and washed with distilled water, 1 L for every 3 theoretical grams of solid obtained. The solid was collected and dried in an oven at 120 °C overnight. The resulting material was then grounded in a mortar to make a fine powder and calcined, increasing the temperature up to 500 °C, 700 °C, and 900 °C with a rate of 2 °C/min and maintaining the set temperature for 2 h.

### 3.3. Synthesis of [NEt_4_][HRu_3_(CO)_11_]

In a steel autoclave equipped with a controller, a mantel, and a thermocouple, RuCl_3_·3H_2_O (2.35 g, 9.00 mmol), NaOMe (2.88 g, 53.3 mmol), and [NEt_4_]Br (0.930 g, 4.45 mmol) were dissolved in 45 mL of MeOH in a glass vessel. After a N_2_ gas conditioning, the autoclave is filled with CO at 60 bar pressure. The reaction proceeded under mechanical stirring at 120 ° C for 18 h. At the end of the reaction the crude, which appeared as a moist reddish solid, was moved into a Schlenk under nitrogen atmosphere. Then, the solvent was removed under reduced pressure, and the residue was washed with H_2_O (2 × 20 mL) and toluene (20 mL) and the product extracted with CH_2_Cl_2_ (40 mL). An amount of 1.89 g of dark red solid identified as [NEt_4_][HRu_3_(CO)_11_] was obtained (yield 85% based on Ru). ^1^H-NMR spectra of the prepared cluster are reported in the Supplementary Information (Figures S11 and S12).

FT-IR (CH_2_Cl_2_, 298 K) νCO: 2083 (w), 2016 (vs), 1987 (s), 1952 (m), 1698 (w) cm^−1^. ^1^H NMR (400 MHz, acetone d6, 298 K) δ: −12.51 ppm.

### 3.4. Catalyst Preparation

The ruthenium carbonyl cluster has been supported on the selected support (with a total nominal metal loading of 1.5 wt%) by wet impregnation protocols. [NEt_4_][HRu_3_(CO)_11_] was dissolved in CH_3_CN, and the resulting solutions were gradually added to the support already placed under nitrogen. The slurry was stirred overnight, then the solvent was removed in vacuo, and the catalyst was treated at 120 °C for 2 h maintaining under N_2_ atmosphere. All the samples were thermally treated in a reductive atmosphere (H_2_/N_2_ 10% *v*/*v*) at 350 ° C, increasing the temperature at a rate of 5 °C/min and maintaining it for 2 h, and a selected sample was also treated at 350 °C in other controlled atmospheres using the same rate of temperature (air and N_2_). Some samples were prepared using metal salt impregnation, and they have been synthesized by incipient wetness impregnation using RuCl_3_·3H_2_O as precursor. The concentration of the precursor solution has been calculated to obtain a final total nominal metal loading such as those of the cluster-derived materials. The impregnated samples were dried at 120 °C in air and thermally treated at 350 °C using the same procedure for the supported cluster catalysts.

### 3.5. Characterization of the Materials

The Mg/Al ratio of the LDH and the basic supports and the ruthenium content of the catalysts were determined by inductively coupled plasma atomic emission spectroscopy (ICP-AES) with a Fisons 3410+ instrument (Fisons Instruments, Valencia, CA, USA). The phase composition of the supports was determined by the Rietveld method from XRD powder patterns recorded with Cu Kα radiation on a diffractometer X’pertPro PANalytical with Bragg/Brentano geometry equipped with a fast X’Celerator detector (Malvern Panalytical, Malvern, UK). The mass loss in the decomposition of the LDHs to Mg(Al)O supports was monitored by thermogravimetric (TG) analysis in a Netzsch STA 449 F3 Jupiter instrument (Netzsch, Selb, Germany). The surface area of the supports was determined by N_2_ sorption at 77 K using a Sorpty 1750 Fison instrument (Fison Instruments, Glasgow, UK) on samples outgassed at 120 °C.

The total basicity and the distribution of basic sites of the supports were determined using a TPD Micromeritics instrument. Generally, 15–20 mg of catalyst were pretreated under He flow at a temperature corresponding to the previous calcination temperature of the material. The sample was cooled down to 40 °C, and CO_2_ (5% in He) was adsorbed for 1 h and purged for 30 min in He flow at the same temperature. CO_2_ desorption was monitored by a thermal ramp of 10 °C/min up to 500 °C and 60 min isotherm at the same temperature.

In order to follow the impregnation process of the metal carbonyl clusters in contact with supports, FTIR spectra in the νCO region were recorded with a PerkinElmer SpectrumOne interferometer in CaF_2_ cells during different steps of the preparation: liquid-phase spectra of the cluster dissolved in acetonitrile and of the cluster solution after 16 h in contact with the support; solid-state spectra of the powders (between NaCl crystals in Nujol) dried at room temperature, after thermal treatment in N_2_ at 120 °C, and after the final thermal treatment at 350 °C in reductive atmosphere (5% H_2_ in N_2_).

Size distribution and dispersion of the metal nanoparticles were evaluated by transmission electron microscopy (TEM) using a TITAN Themis 300 S/TEM microscope (FEI manufacturing, Hillsboro, OR, USA) or a TEM/STEM FEI TECNAI F20 microscope (FEI manufacturing, Hillsboro, OR, USA) at 200 keV. A drop of ultrasonicated suspension of the samples in ethanol was deposited on a quantifoil-carbon film supported by a Cu grid, and the preparation was dried at 120 °C.

### 3.6. Catalytic Tests

The catalytic tests were carried out in a 100 mL Amar autoclave with a mechanical stirrer (0–1500 rpm). Standard reactions were conducted at 110 °C, for 4 h, maintaining the stirring rate at 1200 rpm. For the reaction solution, 55.5 mg of HMF were dissolved in 25 mL of distilled water with the necessary amount of catalyst to have a molar ratio of HMF:metal equal to 1:0.01 and a homogeneous base when employed. The solution was added to the reactor, then the autoclave was purged with 10 bar of oxygen 3 times and pressurized at 10 bar (or more when necessary). The reaction time started (t = 0) when the set-point temperature was reached. The temperature within the system was monitored with a thermocouple, connected to a control unit. Once the set reaction time was over, the autoclave was rapidly cooled down using an ice bath and depressurized once the pressure inside had dropped to the initial loaded value. The reaction mixture was recovered with distilled water to a final volume of 100 mL. Once the solution was centrifuged in order to remove the solid catalyst, a sample of the reaction solution was collected and diluted 1:10 ratio. Analysis of the reaction mixture was carried out using an HPLC instrument (Agilent Technologies 1260 Infinity, Agilent Technologies, Santa Clara, CA, USA), with a DAD UV–Vis detector, equipped with a Biorad AMINEX HPX 87H column (300 mm × 7.8 mm, Bio-Rad Laboratories, Inc., Hercules, CA, USA), ideal for the separation of organic acids, using 0.005 M H_2_SO_4_ as eluent. To calculate the concentration of each reactive species present in the reaction (HMF, HMFCA, FFCA, FDCA), an external calibration curve prepared using reference commercial samples was used.

## 4. Conclusions

The use of metal carbonyl clusters is a promising way to obtain Ru-based catalysts through the impregnation of [HRu_3_(CO)_11_]^−^ on supports with different properties (TiO_2_ and Mg/Al-based materials). The activity of the prepared catalysts was evaluated in the selective HMF oxidation to produce FDCA and the results obtained using these catalysts prepared with a methodology not yet tested for Ru catalysts for this reaction, were compared with those of more common systems prepared through metal salt (RuCl_3_·3H_2_O) impregnation. The technique employed allows the formation of small nanoparticles with a mean diameter around 1 nm well-dispersed on both types of support used. Smaller particles were formed on Mg(Al)O supports, correlated to the interaction between the cluster and the support monitored by FT-IR. The cluster-derived samples showed enhanced activity on all supports compared to the catalysts by salt impregnation. Indeed, both the HMF conversion and FDCA selectivity increased by more than 20% when the cluster was employed. The differences in the catalyst activity can be related to the formation of smaller and more active nanoparticles when the clusters were used. The experiments conducted using two different kinds of support, TiO_2_ and Mg/Al-based materials, showed the possibility of tuning the catalytic properties of the prepared systems in order to conduct the HMF oxidation in base-free conditions. A different behavior of the ruthenium catalyst on different supports was observed. In particular, the initial HMF oxidation and the FFCA formation occurred faster when a homogeneous base and TiO_2_ were employed, but the final FFCA oxidation to FDCA was faster when support with basic properties was employed, leading to a more selective system to FDCA. Finally, the preparation of Mg/Al-based support with different Mg/Al molar ratios and calcined at different temperatures has allowed preparation of supports with different basic site distribution, as monitored by CO_2_-TPD. This has allowed a definite correlation between basic strength and selectivity in FDCA, highlighting that a more significant number of weak basic sites can enhance the reaction, whereas stronger basic sites tend to reduce the catalyst selectivity.

## Figures and Tables

**Table 1:** XRD cell parameters of the materials as a function of the calcination temperature.

	LDH a (Å)	LDH c (Å)	Mg(Al)O a (Å)			Spinel *w*/*w* Fraction
			Calcination Temperature (°C)			
			500	700	900	900
Mg4Al	3.072	23.461	4.177	4.177	-	-
Mg3Al	3.061	23.297	4.177	4.180	4.199	0.179
Mg2Al	3.045	22.840	4.178	4.182	4.194	0.267

**Table 2:** Surface area and Scherrer crystallite size of the materials calcined at different temperature levels.

	Surface Area (m^2^ g^−1^)			Mg(Al)O Size (nm)			Spinel Size nm
Calcination Temperature (°C)	500	700	900	500	700	900	900
Mg4Al	115	157	-	5.0	5.7	-	-
Mg3Al	111	195	122	4.0	4.8	7.2	9.8
Mg2Al	91	141	101	3.8	4.1	7.6	9.2

**Table 3:** Amount of adsorbed CO_2_ and distribution by type of basic site.

Sample	Calcination Temperature	Total CO_2_		CO_2_ Fraction		
	°C	μmol g^−1^	μmol m^−2^	w.b.s. *	m.s.b.s. *	p.a.b.s. *
TiO_2_	500	15	0.19	0.64	0.34	0.02
Mg2Al	500	290	3.19	0.475	0.402	0.123
Mg3Al		320	2.88	0.522	0.441	0.037
Mg4Al		240	2.09	0.426	0.553	0.021
Mg2Al	700	130	0.92	0.409	0.585	0.006
Mg3Al		250	1.28	0.278	0.559	0.163
Mg4Al		120	0.76	0.373	0.430	0.198
Mg2Al	900	130	1.29	0.522	0.353	0.125
Mg3Al		210	1.72	0.303	0.440	0.256

* w.b.s.: weak basic site; m.s.b.s.: medium-strong basic sites; p.a.b.s.: poorly accessible basic sites.

**Table 4:** Mean Ru-particle size (Figure S9), experimental metal loading, and total basicity (calculated through TPD-CO_2_ analysis) of the prepared Ru-based catalysts.

Catalyst	Mean Particle Size (nm)	Experimental Metal Loading (%)	Total Basicity (mmol CO_2_/g)
Ru/TiO_2_-C ^a^	1.4 ± 0.4	1.5	0.02
Ru/TiO_2_-S ^b^	1.8 ± 0.4	1.4	0.02
Ru/Mg3Al-500-C	1.2 ± 0.3	1.5	0.32

^a^ Catalysts prepared through cluster decomposition. ^b^ Catalysts prepared through salt decomposition.

**Table 5:** Blank tests made on an aqueous solution of HMF and catalytic supports in the absence of metal NPs. Operative conditions: time 4 h, temperature 110 °C, O_2_ pressure 10 bar; when base was used: molar ratio NaHCO_3_/HMF = 2.

Catalyst	HMF Conversion (%)	HMFCA Yield (%)	DFF Yield (%)	FFCA Yield (%)	FDCA Yield (%)	Others Yield (%)
No support, base-free	4	-	-	-	-	4
No support with base	26	2	-	5	1	18
TiO_2_, base-free	8	-	-	-	-	8
TiO_2_ with base	35	5	-	7	2	21
Mg3Al-500	26	2	5	5	2	12
Mg3Al-700	27	2	4	3	1	17
Mg3Al-900	28	2	4	4	0	18

**Table 6:** Catalytic tests employing reaction intermediates (HMFCA, DFF, and FFCA) as feed reagents and using Ru/TiO_2_-C as catalyst. Operative conditions: time 4 h, temperature 130 °C, O_2_ pressure 10 bar, molar ratio reagent/total metal = 100, molar ratio NaHCO_3_/reagent = 2.

Entry	Reagent	Conversion (%)	Yield FFCA (%)	Yield FDCA (%)	Others (%)
1	HMFCA	39	25	8	6
2	DFF	100	78	19	3
3	FFCA	26	-	22	4

## Data Availability

The original contributions presented in this study are included in the article/Supplementary Material. Further inquiries can be directed to the corresponding author.

## Supplementary Materials

The following supporting information can be downloaded at: https://www.mdpi.com/article/10.3390/molecules30102120/s1, Figure S1: XRD pattern and TPD-CO_2_ analysis of commercial DT51 TiO_2_; Figure S2: XRD patterns of all the prepared materials calcined at different temperature and with different Mg:Al molar ratios: 2 (A), 3 (B), and 4 (C); Table S1: List of all the Ru-based prepared catalysts using metal carbonyl clusters and metallic salts as active phase precursors along with their experimental Mg:Al molar ratio and experimental metal loading; Figure S3: TGA profiles obtained from the analyses of the three LDHs with the different Mg:Al molar ratios of 2, 3, and 4; Figure S4: TPD-CO_2_ profiles of Mg/Al-based materials and their deconvolution; Figure S5: FTIR spectra recorded during the [HRu_3_(CO)_11_]^−^ impregnation protocol over Mg/Al-based materials with a molar ratio of 2 and calcined at 500 °C (A), 700 °C (B), and 900 °C (C); Figure S6: FTIR spectra recorded during the [HRu_3_(CO)_11_]^−^ impregnation protocol over Mg/Al-based materials with a molar ratio of 6 and calcined at 500 °C (A), 700 °C (B), and 900 °C (C); Figure S7: FTIR spectra recorded during the [HRu_3_(CO)_11_]^−^ impregnation protocol over Mg/Al-based materials with a molar ratio of 4 and calcined at 500 °C (A), and 700 °C (B); Figure S8: TEM images collected on the Ru/TiO_2_ catalyst prepared through incipient wetness impregnation of RuCl_3_·3H_2_O; Figure S9: Nanoparticles distribution of the Ru/TiO_2_-C (A), Ru/TiO_2_-S (B), and Ru/Mg(Al)O prepared with the Mg/Al-based support with a Mg:Al molar ratio of 3 and calcined at 500 °C (C); Table S2: Comparison of the catalyst surface area before and after the deposition of Ru nanoparticles; Figure S10: Effect of the stirring rate using Ru/TiO_2_ catalyst (A) with a focus on the HMF conversion (B); Figure S11: ^1^H-NMR spectrum of [NEt_4_][HRu_3_(CO)_11_] in acetone-d^6^; Figure S12: Hydride region of ^1^H-NMR spectrum of [NEt_4_][HRu_3_(CO)_11_] in acetone-d^6^.

## Abbreviations

The following abbreviations are used in this manuscript:

HMF	5-hydroxymethylfurfural
HMFCA	5-hydroxymethyl-2-furancarboxylic acid
DFF	2,5-diformylfuran
FFCA	5-formyl-2-furancarboxylic acid
FDCA	2,5-furandicarboxylic acid
MCC	Metal carbonyl clusters
NP	Nanoparticle
LDH	Layer double hydroxide
w.b.s	weak basic sites
m.s.b.s.	medium and strong basic sites
p.a.b.s.	poorly accessible basic sites
