# Supplementary material for: Preparation of Ru-Based Systems Through Metal Carbonyl Cluster Decomposition for the Base-Free 5-Hydroxymethylfurfural (HMF) Oxidation
Source: Molecules. 2025 May 10;30(10):2120. doi: 10.3390/molecules30102120 (PMC12114423; doi:10.3390/molecules30102120)
Supplement: Supplementary file 1 [file molecules-30-02120-s001.zip › molecules-3584798-supplementary.pdf]

Preparation of Ru-based systems through metal carbonyl cluster decomposition for the base-free 5-hydroxymethylfurfural (HMF) oxidation

Francesca Liuzzi [1], Francesco Di Renzo[2], Cristiana Cesari [1], Alice Mammi [1], Lorenzo Monti [1], Stefano Zacchini [1], Giuseppe Fornasari [1], Nikolaos Dimitratos [1] and Stefania Albonetti [1,*]

[1] Department of Industrial Chemistry, C3-Centre for Chemical Catalysis, Alma Mater Studiorum – University of Bologna, Bologna, Italy; stefania.albonetti@unibo.it

[2] ICGM, Université de Montpellier-CNRS-ENSCM, Montpellier 34293, France; francesco.di-renzo@umontpellier.fr

* Correspondence: stefania.albonetti@unibo.it

Supporting information

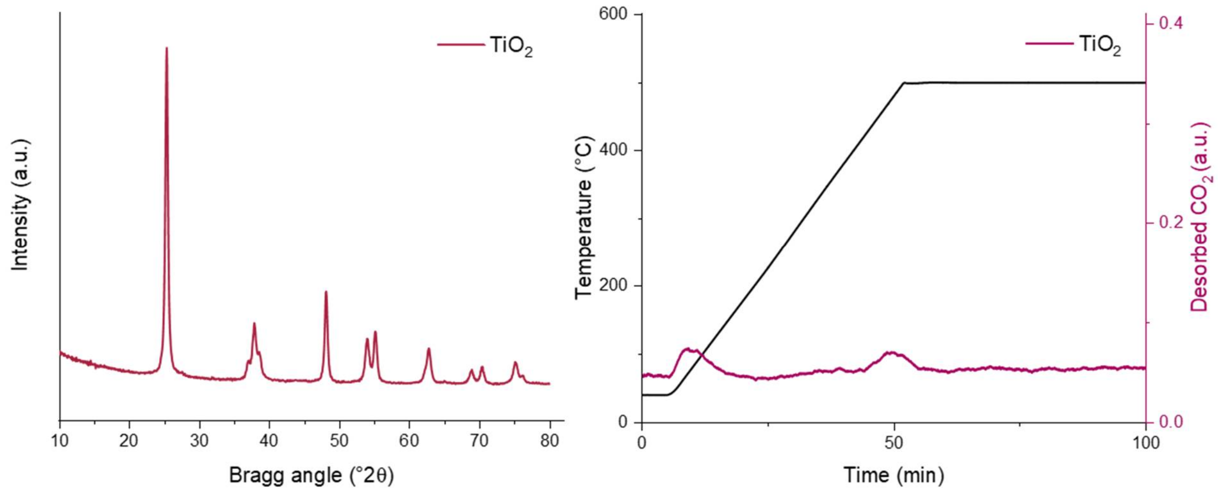

Figure S1. XRD pattern and TPD-CO_2 analysis of commercial DT51 TiO_2.

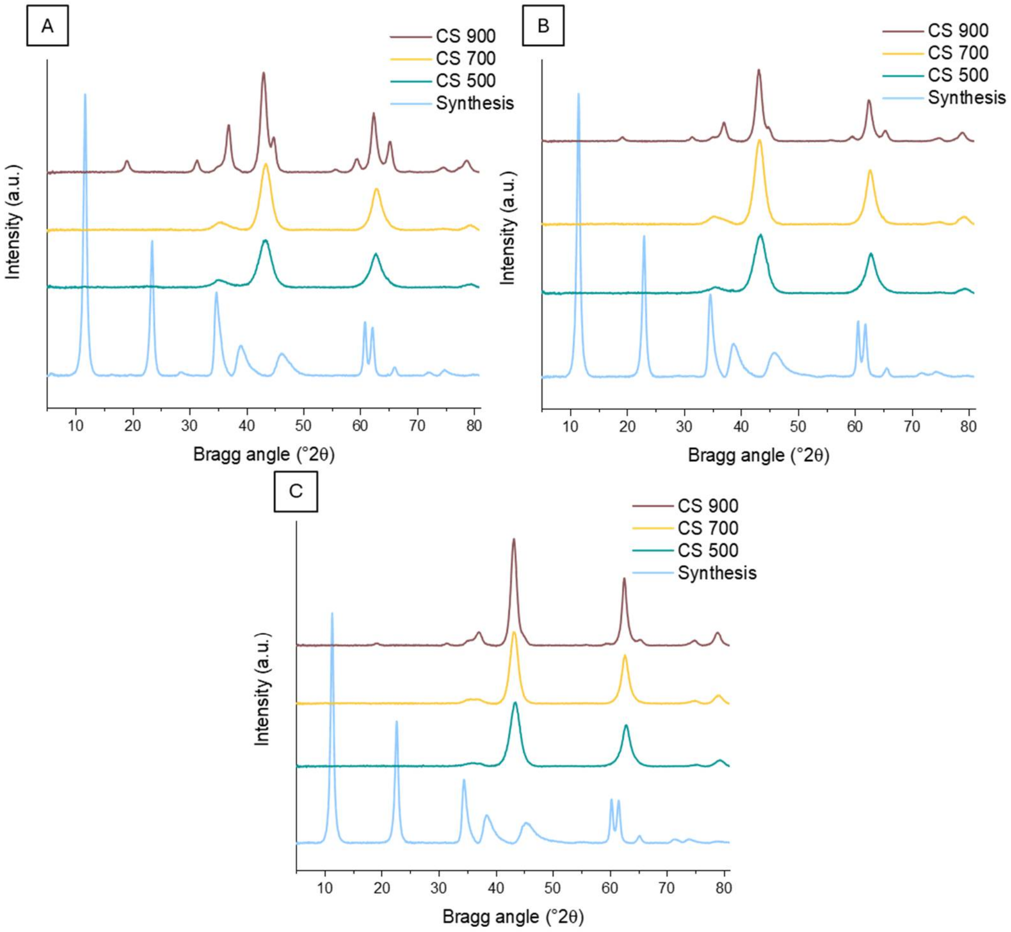

Figure S2. XRD patterns of all the prepared materials calcined at different temperature and with different Mg:Al molar ratios: 2 (**A**), 3 (**B**), and 4 (**C**).

Table S1. List of all the Ru-based prepared catalysts using metal carbonyl clusters and metallic salts as active phase precursors along with their experimental Mg:Al molar ratio and experimental metal loading.

**Catalyst**	**Active phase precursor**	**Experimental Mg:Al molar ratio**	**Experimental metal loading (%)**
Ru/TiO_2-H_2-C	$[HRu_3(CO)_{11}]^-$	-	1.5
Ru/TiO_2-H_2-S	$RuCl_3$*$3H_2O$	-	1.4
Ru/Mg2Al-500-C	$[HRu_3(CO)_{11}]^-$	2.2	1.5
Ru/Mg2Al-700-C	$[HRu_3(CO)_{11}]^-$	2.2	1.4
Ru/Mg2Al-900-C	$[HRu_3(CO)_{11}]^-$	2.2	1.5
Ru/Mg3Al-500-C	$[HRu_3(CO)_{11}]^-$	3.1	1.5
Ru/Mg3Al-700-C	$[HRu_3(CO)_{11}]^-$	3.2	1.5
Ru/Mg3Al-900-C	$[HRu_3(CO)_{11}]^-$	3.1	1.4
Ru/Mg4Al-500-C	$[HRu_3(CO)_{11}]^-$	4.0	1.3
Ru/Mg4Al-700-C	$[HRu_3(CO)_{11}]^-$	4.0	1.2

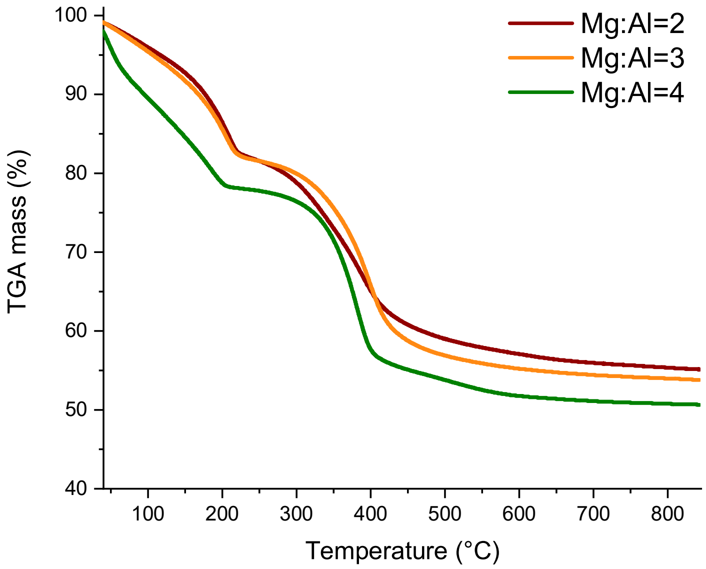

Figure S3. TGA profiles obtained from the analyses of the three LDHs with the different Mg:Al molar ratios of 2,3 and 4.

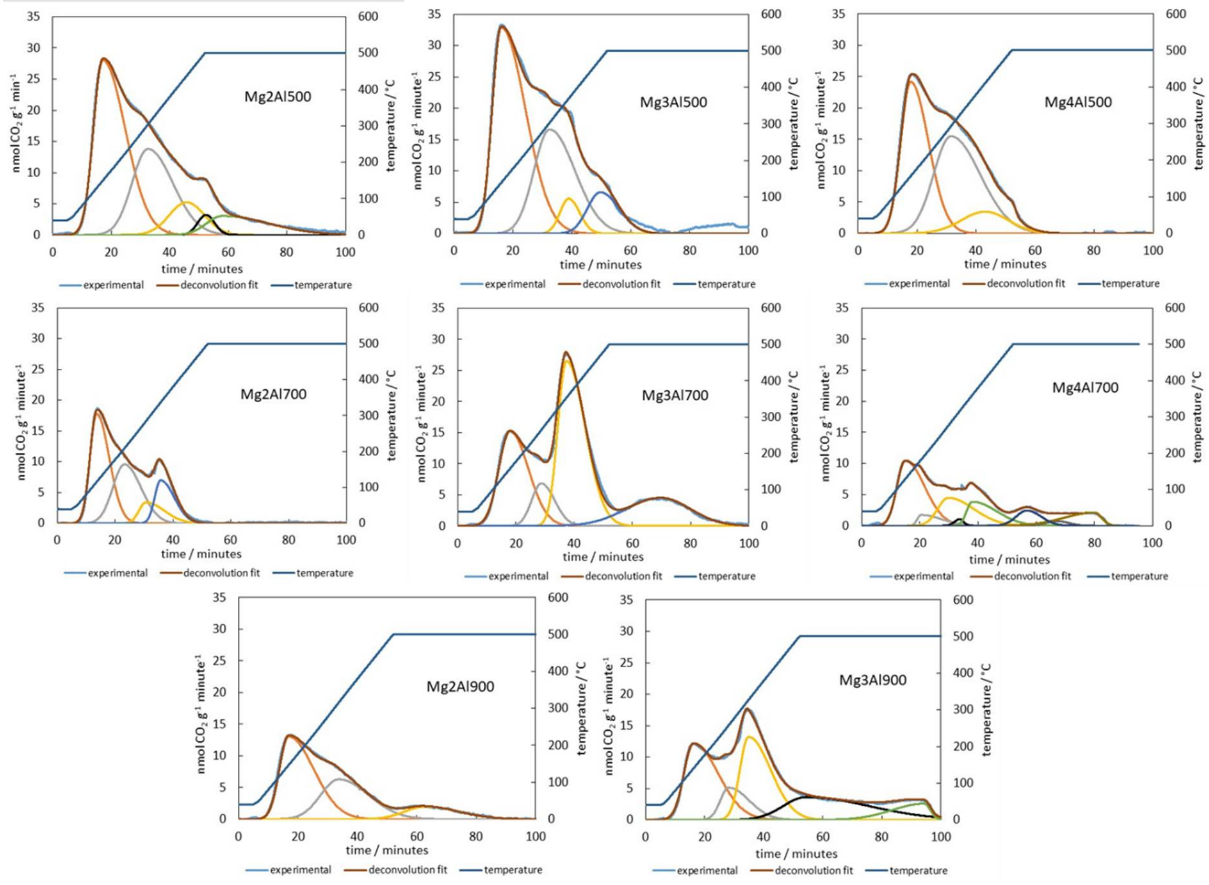

Figure S4. TPD-CO_2 profiles of Mg/Al-based materials and their deconvolution.

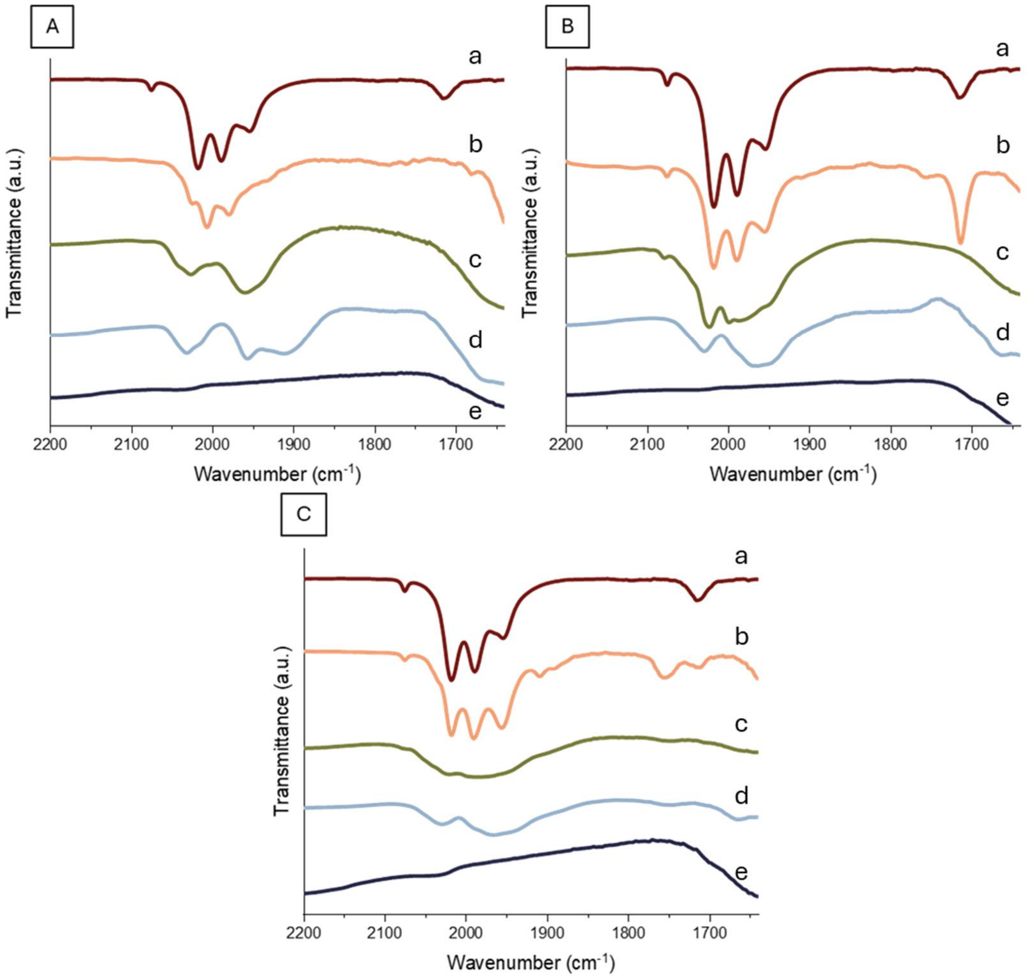

Figure S5. FTIR spectra recorded during the $[HRu_3(CO)_{11}]^-$ impregnation protocol over Mg/Al-based materials with a molar ratio of 2 and calcined at 500 °C (**A**), 700°C (**B**), and 900 °C (**C**). Each spectrum was recorded during different steps of the preparation. Cluster dissolved in CH_3CN (a), cluster and MgO suspension in CH_3CN (b), dried catalyst powder (c), catalyst powder after thermal treatment at 120 °C under N_2 (d), and catalyst powder after a second thermal treatment at 350 °C under reductive atmosphere (e).

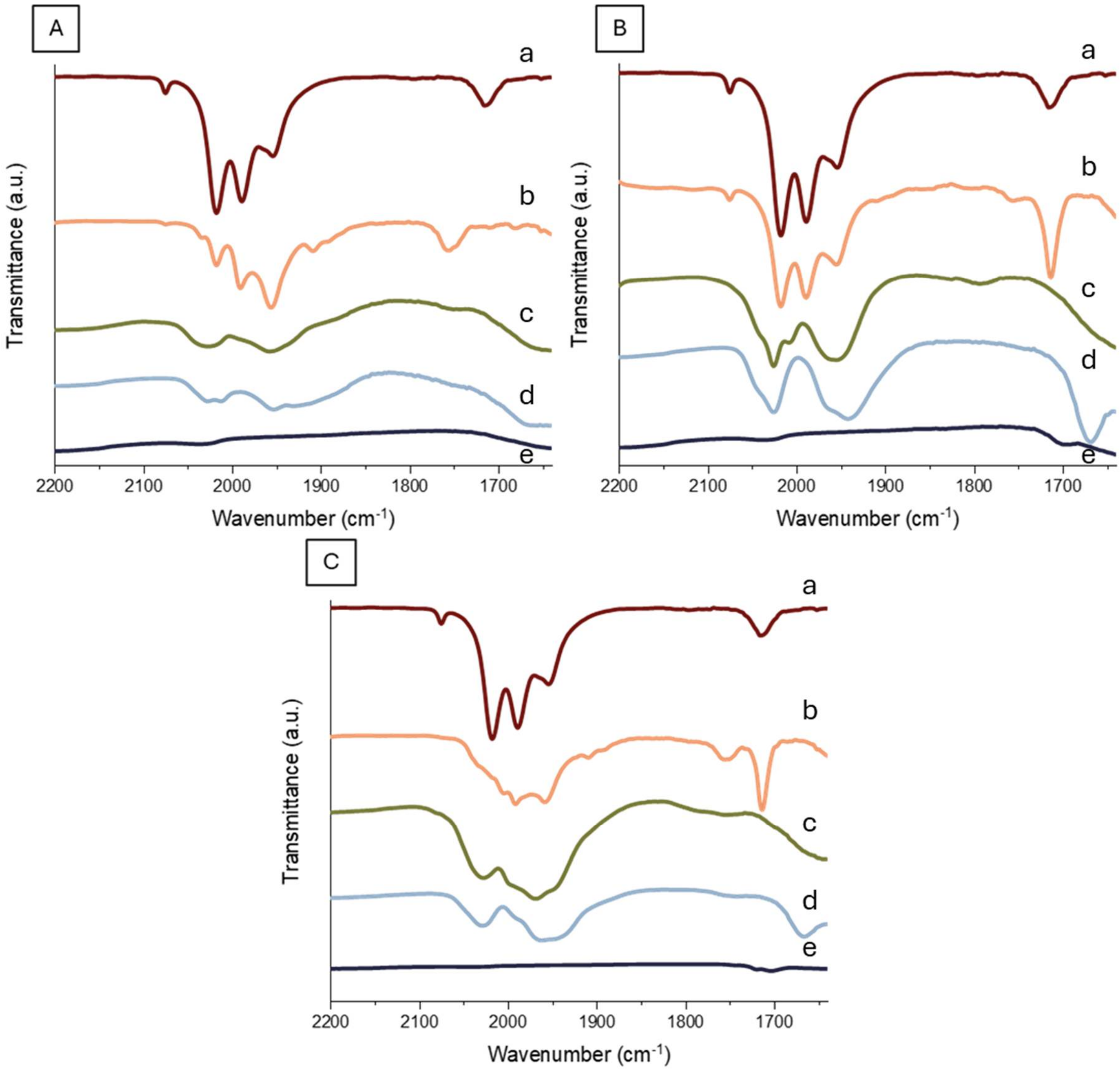

Figure S6. FTIR spectra recorded during the $[HRu_3(CO)_{11}]^-$ impregnation protocol over Mg/Al-based materials with a molar ratio of 3 and calcined at 500 °C (**A**), 700°C (**B**), and 900 °C (**C**). Each spectrum was recorded during different steps of the preparation. Cluster dissolved in CH_3CN (a), cluster and MgO suspension in CH_3CN (b), dried catalyst powder (c), catalyst powder after thermal treatment at 120 °C under N_2 (d), and catalyst powder after a second thermal treatment at 350 °C under reductive atmosphere (e).

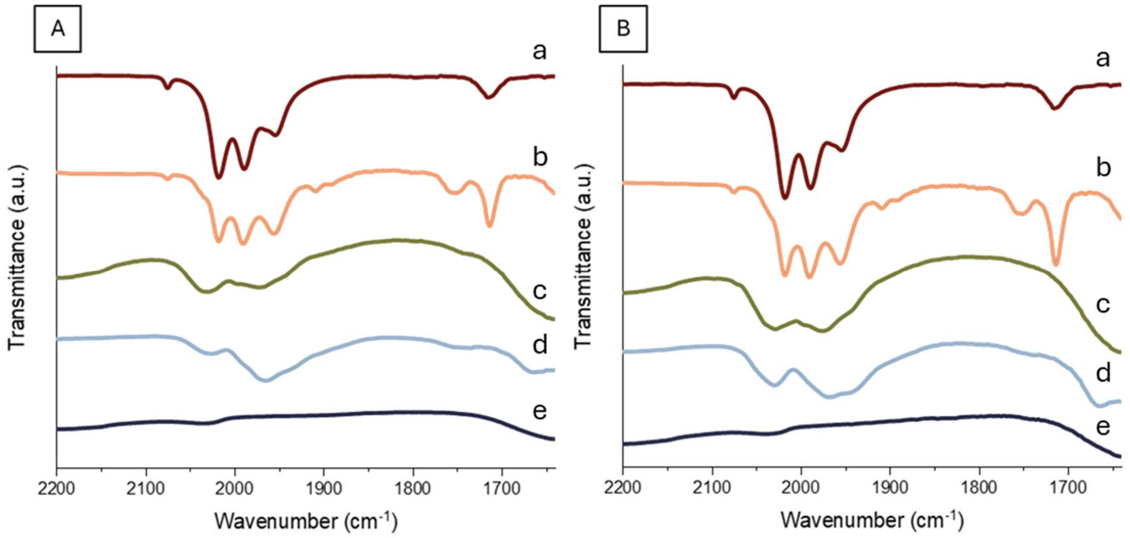

Figure S7. FTIR spectra recorded during the $[HRu_3(CO)_{11}]^-$ impregnation protocol over Mg/Al-based materials with a molar ratio of 4 and calcined at 500 °C (**A**), and 700°C (**B**). Each spectrum was recorded during different steps of the preparation. Cluster dissolved in CH_3CN (a), cluster and MgO suspension in CH_3CN (b), dried catalyst powder (c), catalyst powder after thermal treatment at 120 °C under N_2 (d), and catalyst powder after a second thermal treatment at 350 °C under reductive atmosphere (e).

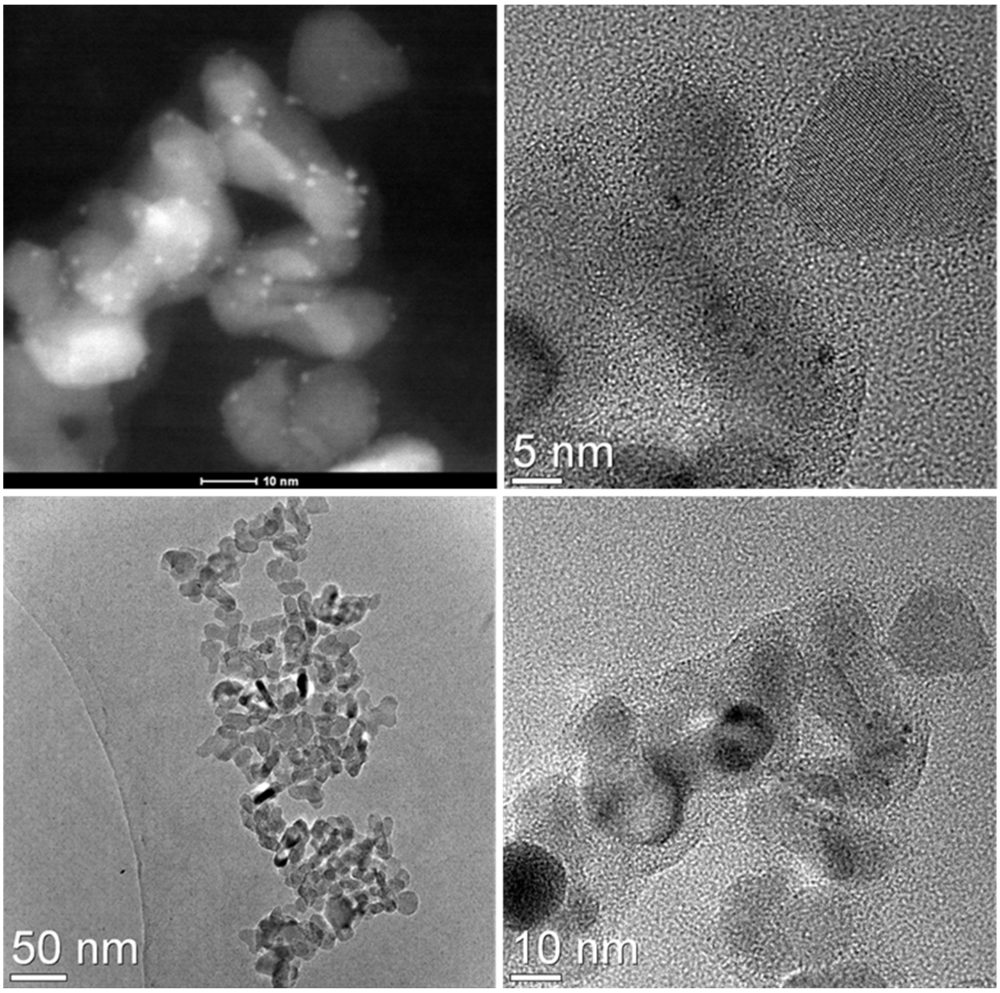

Figure S8. TEM images collected on the Ru/TiO_2 catalyst prepared through incipient wetness impregnation of $RuCl_3$*$3H_2O$. Some Agglomeration of Ru (darker zones) can be noted from the images.

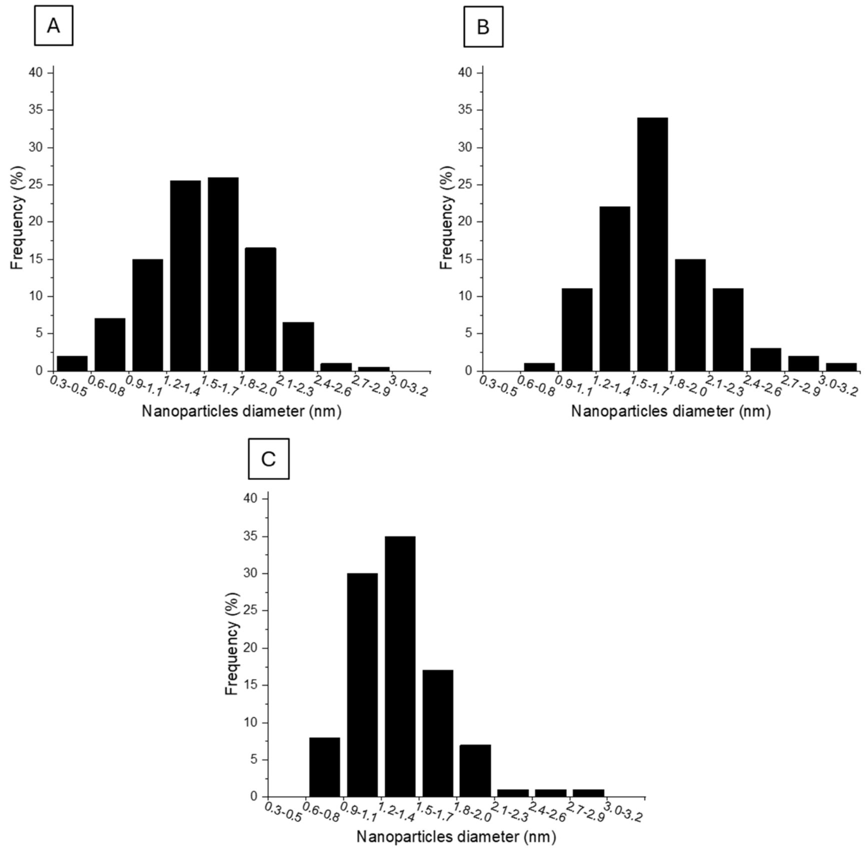

Figure S9. Nanoparticles distribution of the Ru/TiO_2-C (**A**), Ru/TiO_2-S (**B**), and Ru/Mg(Al)O prepared with the Mg/Al-based support with a Mg:Al molar ratio of 3 and calcined at 500 °C (**C**).

Table S2. Comparison of the catalyst surface area before and after the deposition of Ru nanoparticles.

**Catalyst**	**Support surface area (m^2g^{-1})**	**Final catalyst surface area (m^2g^{-1})**
Ru/TiO_2-C	69	71
Ru/TiO_2-S	69	62
Ru/Mg3Al-500-C	111	115

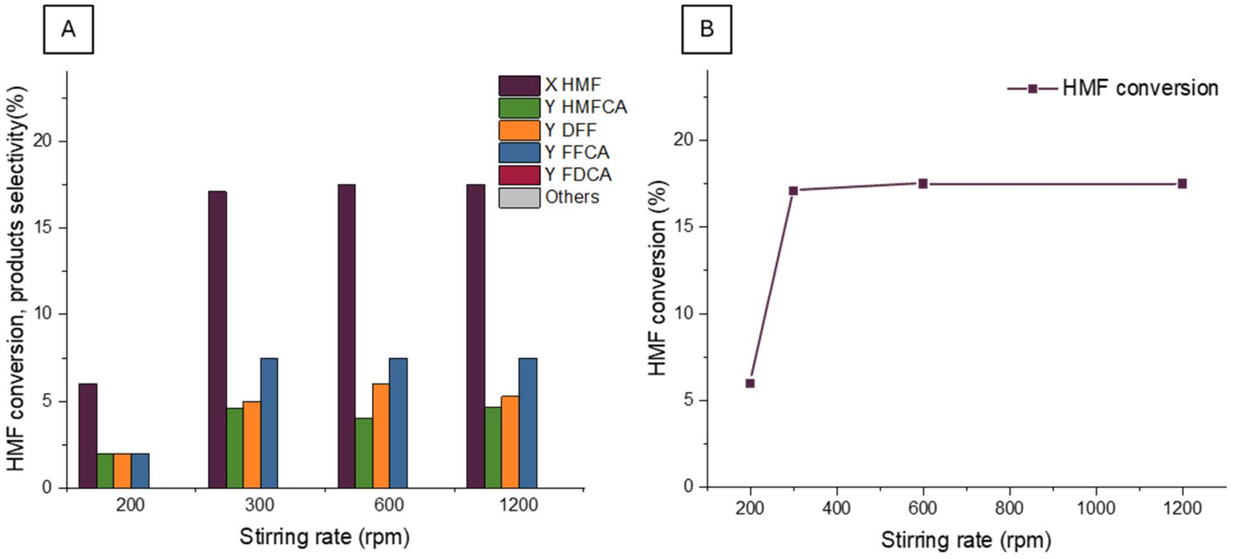

Figure S10. Effect of the stirring rate using Ru/TiO_2 catalyst (**A**) with a focus on the HMF conversion (**B**). Operative conditions: time 10 min, temperature 110 °C, O_2 pressure 10 bar, molar ratio HMF/total metal = 100, molar ratio $NaHCO_3$/HMF = 2.

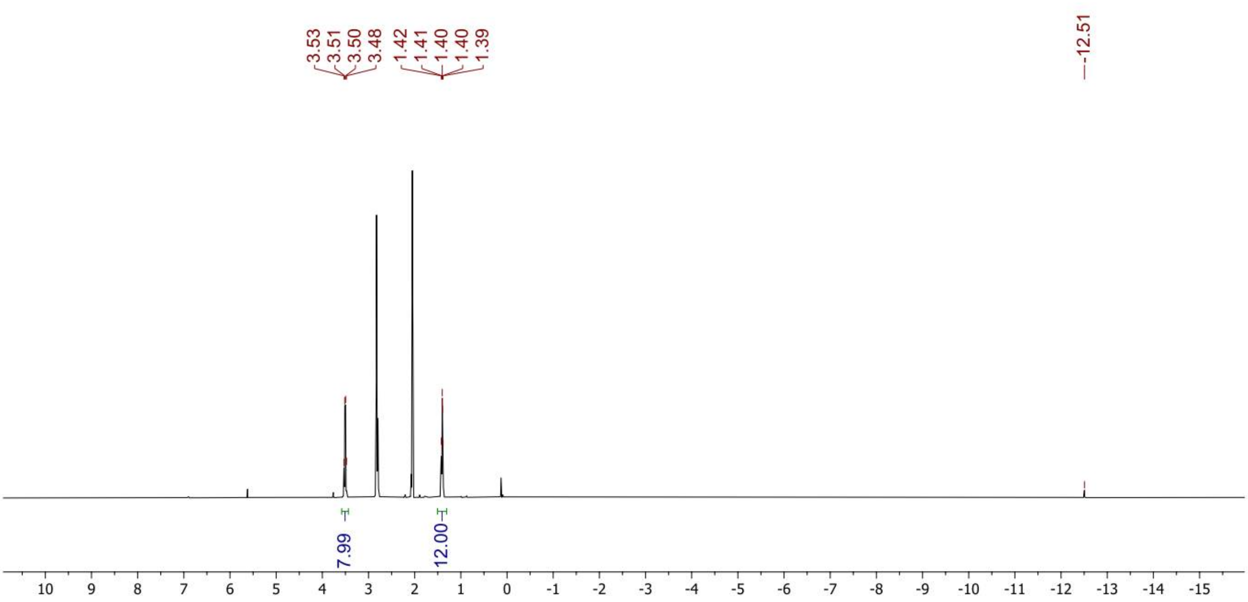

Figure S11. ^{1}H-NMR spectrum of $[NEt_4][HRu_3(CO)_{11}]$ in acetone-d^6.

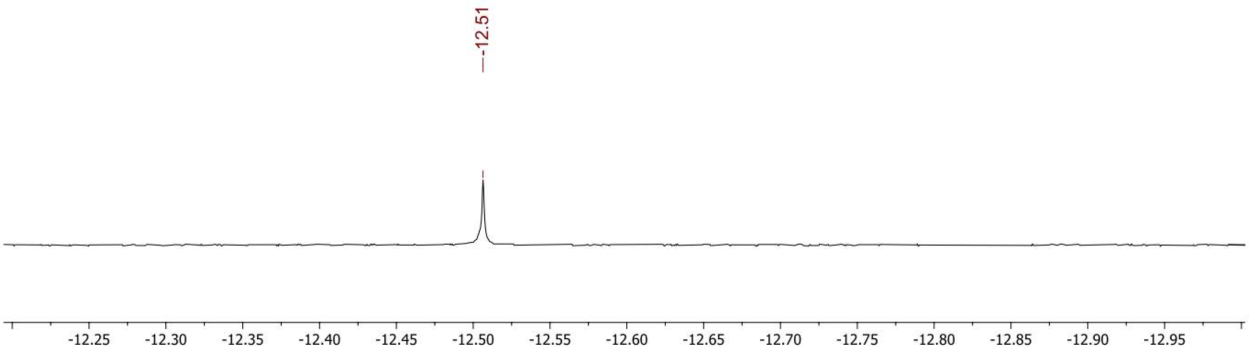

Figure S12. Hydride region of ^{1}H-NMR spectrum of $[NEt_4][HRu_3(CO)_{11}]$ in acetone-d^6.
